# For Me or Against Me? Reactions to AI (vs. Human) Decisions That Are Favorable or Unfavorable to the Self and the Role of Fairness Perception

**DOI:** 10.1177/01461672241288338

**Published:** 2024-10-24

**Authors:** Jungmin Choi, Melody M. Chao

**Affiliations:** 1 Judge Business School, University of Cambridge, Cambridge, UK; 2 Department of Management, Hong Kong University of Science & Technology, Clear Water Bay, Kowloon, Hong Kong SAR

**Keywords:** artificial intelligence, decision-making, fairness perception, fairness heuristic theory

## Abstract

Public reactions to algorithmic decisions often diverge. While high-profile media coverage suggests that the use of AI in organizational decision-making is viewed as unfair and received negatively, recent survey results suggest that such use of AI is perceived as fair and received positively. Drawing on fairness heuristic theory, the current research reconciles this apparent contradiction by examining the roles of decision outcome and fairness perception on individuals’ attitudinal (Studies 1–3, 5) and behavioral (Study 4) reactions to algorithmic (vs. human) decisions. Results from six experiments (N = 2,794) showed that when the decision was unfavorable, AI was perceived as fairer than human, leading to a less negative reaction. This heightened fairness perception toward AI is shaped by its perceived unemotionality. Furthermore, reminders about the potential biases of AI in decision-making attenuate the differential fairness perception between AI and human. Theoretical and practical implications of the findings are discussed.

Whether in allocating resources, evaluating performance, or enforcing disciplinary actions, organizations often make high-stakes decisions that significantly impact their employees. In these situations, organizations are typically concerned with employees’ perception of fairness, as this perception plays a crucial role in shaping their reactions. When employees perceive unfairness, they are more likely to react negatively, exhibiting behaviors such as withdrawal from work (Ball et al., 1993), retaliation (Wanberg et al., 1999), and lower commitment (Shapiro & Kirkman, 1999). This subjective sense of fairness, which can profoundly affect both interpersonal and organizational outcomes, is often shaped by the characteristics of decision agents involved in the decision-making process (Patient & Skarlicki, 2010; Richter et al., 2018) along with the nature of the decision outcomes (Ditto et al., 2003).

In today’s organizations, decisions are made not only by traditional decision agents such as managers and supervisors but also by artificial intelligence (AI), a set of technologies that enable algorithms to learn autonomously from data and generate solutions (Google, 2023a).^1^ For example, AI is used to assess job candidate competencies and automatically reject those who are considered inadequate (Murad, 2021). AI also determines schedules and target productivity levels for workers (Solon et al., 2022). Moreover, an AI system has allegedly generated termination decisions for employees who did not reach the expected level of performance (Lecher, 2019).

Despite AI’s increasing role in making high-stakes decisions for organizations, public discourse on whether AI as a decision agent is perceived as more or less fair than a human decision agent is conflicted. On the one hand, numerous media outlets have covered high-profile cases in which the use of AI in organizational decision-making was seen as unfair and received negative press. For example, in early 2023, a job candidate filed a lawsuit against a cloud firm, claiming that the company’s AI-based hiring software discriminated against black and older applicants and led to unfair rejection of qualified applicants (Gilbert, 2023; Yates, 2023). In November 2022, warehouse workers from a multinational technology company participated in protests, accusing the company of unfair labor practices, which included the use of algorithms to determine the target productivity level, inducing unreasonable pressure on the workers (Sherter, 2022; Solon et al., 2022). Furthermore, over the past few years, multiple lawsuits were filed against transportation companies by former drivers who claimed that they were dismissed unfairly by algorithms ( Butler, 2021; Russon, 2020; Prosser, 2020). Given such media coverage of negative reactions to algorithmic decisions, the general public would arguably be skeptical about the fairness of AI as a decision agent.

On the other hand, contrasting to the high-profile stories covered in the news media, results from recent surveys suggest that people generally perceive the use of AI in making decisions as fair. According to a survey conducted by Pew Research Center in 2022, 54% of the 11,004 respondents who view racial and ethnic discrimination as an issue during the hiring process and performance evaluations believed that the use of AI would result in fairer practices, while only 13% responded that AI would make the issue worse. The same survey further noted that 48% of the respondents believed that AI would promote equality in the hiring process, compared with 15% who were skeptical of AI (Rainie et al., 2023). These statistics suggest that AI is often seen as a fair decision agent, particularly in high-stakes contexts (e.g., job application rejection, negative performance review). Moreover, in a 2023 survey (Gillespie et al., 2023), only 22% of the 17,000 respondents reported feeling uncomfortable with using AI to inform organizational decision-making. Most of the respondents reported feeling comfortable (50%) or neutral (28%) with such use of AI.

In sum, the observations of the public discourses suggest that there could be diverging perceptions of fairness and reactions to AI as a decision-making agent, calling for a systematic examination of a factor(s) that may play a crucial role in shaping the perception of fairness and reaction toward AI. Thus, through the lens of fairness heuristic theory (Lind, 2001; van den Bos et al., 1997), the current research aims to reconcile this inconsistency by examining how the perceived fairness of the decision agent (AI vs. human) might be influenced by the decision outcome (favorable vs. unfavorable) and the downstream attitudinal and behavioral implications of fairness perceptions.

## Theoretical Background and Hypotheses

### Fairness Heuristic Theory

According to the fairness heuristic theory, organizational members form a global perception of fairness toward an entity and use the fairness perception as cognitive “shortcuts” or heuristics that guide their subsequent attitudes and behaviors (Lind, 2001; van den Bos et al., 1997). Fairness heuristic theory argues that people in an organizational context often face *the fundamental social dilemma*, in which the contribution of their personal resources can secure their standing and sense of belonging in the organization but at the same time expose them to the risks of being exploited. Thus, to determine whether it is worth investing their resources in an organization, individuals formulate fairness heuristics based on readily available and interpretable information (van den Bos et al., 1997); higher fairness perception would mitigate concerns about potential exploitation, leading employees to be more willing to invest personal resources in the organization (Lind, 2001).

In formulating the fairness heuristic, organizational members often consider the decision agents involved in the decision-making process. Specifically, people can have differential fairness perceptions toward the same decision depending on the attributes of the decision agent (Choi, 2008; Hollensbe et al., 2008); decision agents with qualities associated with fairness are likely to be perceived as fairer. Extant studies on algorithmic decision-making have highlighted the significant role of decision agents in shaping fairness perceptions, showing that individuals hold differential fairness perceptions depending on whether the decision agent is AI or human. However, the evidence from the literature is mixed (Köchling & Wehner, 2020). Whereas one line of studies found that AI (vs. human) is perceived as less fair (Langer et al., 2019; Lee, 2018; Newman et al., 2020), another line of studies found evidence for higher fairness perception toward AI (vs. human) decision agents (Bai et al., 2022; Marcinkowski et al., 2020). An interesting aspect of these divergent findings is that the same attribute of AI can lead to higher or lower fairness perception compared with humans. For instance, AI’s perceived lack of ability to interpret qualitative information (Newman et al., 2020) and to make subjective and intuitive judgments (Lee, 2018) can lower the perceived fairness of AI (vs. human) because it fails to take circumstantial information into account and consequently appears impersonal and rigid. Such lack of ability, however, can at the same time make AI seen as fairer than human by making algorithmic judgment seem objective, consistent, and free of personal biases (Bai et al., 2022; Bonezzi & Ostinelli, 2021; Jago & Laurin, 2022). This suggests that the perceived fairness of a decision agent can vary depending on the context, which in turn influences reactions to algorithmic versus human decisions (Castelo et al., 2019). In sum, the existing literature suggests that to advance our understanding of the psychology involved in algorithmic decision-making, it is important to contextualize our understanding of fairness perception by taking the context and the characteristics of the decision agents into consideration.

Past research on organizational justice suggests that the favorability of a decision to the self may be a crucial contextual factor that interplays with the decision agent to form overall fairness perception. Generally, decisions that are favorable to the individual tend to heighten perceived fairness, whereas unfavorable decisions lower fairness perception (Brockner & Wiesenfeld, 1996; Conlon & Fasolo, 1990; Conlon & Ross, 1993). However, when decisions are unfavorable, individuals’ reactions may differ depending on whether they perceive the decision-making process as fair (Brockner et al., 1994; van den Bos et al., 1997). Given that decision agents play an essential role in the decision-making process, their impartiality is scrutinized in the face of an unfavorable decision (Jones & Skarlicki, 2013; Kouchaki et al., 2015). Taken together, the decision agent and decision outcome favorability jointly influence the formulation of the fairness heuristic. The current study examines how these two factors interact to shape people’s perceptions of fairness in algorithmic decision-making.

### Perceived Fairness of AI: Decision Favorability and Decision Agent

Drawing from the fairness heuristic theory, we posit that, in the face of a favorable decision, individuals would form a fairness perception similarly toward an algorithmic (vs. human) decision agent. Specifically, when the decision is favorable to the self, people are more likely to perceive fairness regardless of whether the decision was made by human or AI. That is, the favorable decision leads one to perceive a sense of fairness heuristically, as recipients of favorable decisions are unlikely to scrutinize characteristics related to the decision further. Extant literature on motivated reasoning is consistent with this argument. It contends that people who face preference-consistent outcomes engage in biased recollection and construction of evidence that supports their preferred conclusions (Ditto et al., 1998) to maintain “an illusion of objectivity” (Pyszczynski & Greenberg, 1987, p. 319). Accordingly, when the decision is favorable, individuals perceive external information that is consistent with their preferred conclusion as more valid (Ditto et al., 2003). As individuals often have an inflated sense of their contributions in a social context (Brockner & Wiesenfeld, 1996; Messick & Sentis, 1979; Ross & Sicoly, 1979), the favorable decision can be seen as a proper outcome for their input, leading to higher fairness perception. Therefore, we posit that individuals would show higher fairness perception when they receive favorable (vs. unfavorable) decisions, regardless of whether the decision agents are AI or human.

When the decision is unfavorable, however, people will look beyond the decision and scrutinize the characteristics of the decision agent. In other words, they are more likely to engage in “effortful cognitive analysis” to examine the validity of unwanted outcomes (Ditto et al., 2003, p. 1121). Thus, when the decision is unfavorable to the self, individuals would closely examine the attributes of the decision agents that shape the decision process and formulate their fairness perception (Leventhal, 1980; Marques et al., 2017). Would AI or human be seen as a fairer decision agent in this case?

We posit that AI is likely to be perceived as fairer than human, as AI is likely to be seen as more impartial with less judgment bias than humans. Existing studies on organizational justice have shown that, in the face of an unfavorable outcome, individuals’ perception of fairness is likely to increase when they perceive that decision agents are free of personal biases (Colquitt et al., 2001). This is because, when individuals make sense of the unfavorable decision, they infer the quality of the treatment they received during the decision-making process by analyzing whether the disadvantageous outcome was due to the decision agent’s subjective judgment (Lind & Tyler, 1988).

Although the academic and professional communities might be highly conscious of the fact that AI can produce biased output due to biases in the input (Akter et al., 2022; Lambrecht & Tucker, 2019), there exist prevailing perceptions of AI that may contribute to higher perceived fairness toward AI (vs. human) among the public. First, there is a prevalent lay belief that AI is an unemotional entity (Jago & Laurin, 2022; Martínez-Miranda & Aldea, 2005). Studies on machine heuristics have shown that computational systems are often perceived to be deprived of “emotional capability,” a core characteristic that defines “humanness” (Gray & Wegner, 2012; Waytz & Norton, 2014). As emotions are commonly seen as a major source of decision bias, the lack of emotion in AI would lead people to perceive AI as fairer than human (Lee, 2018; Sundar & Nass, 2001). Moreover, AI is often depicted as an objective entity (Gillespie, 2014). Given that AI is guided by a set of logic that likely yields consistent outcomes (Korteling et al., 2021), this leaves little room for individuals to engage in counterfactual thinking about potential alternative outcomes (Folger & Cropanzano, 2001; Ganegoda & Folger, 2015). Accordingly, individuals will be less likely to think about the possibility of the decisions being different or being influenced by bias when the decision agent is AI (vs. human). Therefore, in the face of unfavorable decisions, individuals would scrutinize the attributes of the decision agent, perceiving AI as fairer than humans.

### Implications of Perceived Fairness

Fairness heuristic theory argues that people’s perception of fairness has important downstream consequences on the extent to which individuals would cooperate or engage with the other parties (Proudfoot & Lind 2015). For example, individuals are more likely to accept an authority’s decision (Lind et al., 1993), engage in cooperative behavior (De Cremer & van Knippenberg, 2003), and commit to the organization (Sweeney & McFarlin, 1993) following dissatisfying decision outcomes when they perceive that the decisions were made using a fair procedure. Furthermore, perceived fairness may signal that the decision agent is trustworthy, and that individuals’ future engagement or efforts will not be exploited (Brockner et al., 2001). In this study, we examine two downstream impacts of fairness perception toward an algorithmic (vs. human) decision agent: (a) whether one would accept the decision outcomes without challenging them—decision acceptance, and (b) whether one would be willing to continue to invest their resources in the organization—future engagement. We argue that, in the face of an unfavorable decision, the higher fairness perception of AI (vs. human) will lead to higher decision acceptance and future engagement. These two downstream consequences are particularly crucial in organization contexts because many resources are often invested in decision-making processes; if a decision is rejected because of fairness concerns, it can be costly for the organization as it might fail to recruit or engage talents (e.g., Murphy, 1986) who would have contributed to organizational performance (Avery et al., 2007) and innovation (Hakanen et al., 2008).

### The Exploratory Role of Learning About AI

Building on these hypothesized effects, the current research explores an additional question: Can learning about AI’s capabilities and biases alter people’s perception of algorithmic fairness? Thus far, we hypothesize that individuals may have a tendency to perceive AI as fairer than humans in the face of unfavorable decisions. However, in recent years, there has been increasing media coverage of cases where AI has produced biased decisions due to biased training data, potentially perpetuating or even exacerbating gender and racial discrimination (Bousquette, 2023; Dastin, 2018; Nicoletti & Bass, 2023). This suggests a discrepancy between the assumed fairness of AI and the reality that AI replicates human biases. The overestimation of the fairness of algorithmic decisions is often due to the lack of awareness of AI biases, causing individuals to unknowingly propagate and accept existing inequalities. Through education, however, it might be possible to attenuate or reverse the effects, thereby preventing the perpetuation of biases.

In line with this idea, existing research has shown that the perception of AI is not static and can evolve with new information and experiences. For example, Jago and Laurin (2022) found that as individuals assume that algorithms are more accurate and unemotional than humans, they prefer to be evaluated by algorithm (vs. human) when they anticipate discrimination; however, when they learn that algorithms are trained based on datasets composed of previous human judgments, they no longer preferred to be evaluated by algorithms. These findings, combined with fairness heuristic theory—which posits that perceptions of fairness can be dynamic (Proudfoot & Lind, 2015)—suggest that individuals can re-evaluate their pre-existing beliefs about algorithmic fairness as well. Therefore, the current research explores whether learning about AI’s potential to replicate human biases can alter individuals’ perceived fairness of AI and their reactions to algorithmic decisions when the outcomes are unfavorable. Examining this question is especially crucial for understanding the malleability of the assumptions about AI that guide people’s perceptions and reactions, providing important implications for the role of AI literacy in engagement with AI technologies.

### Summary

**Figure 1.**
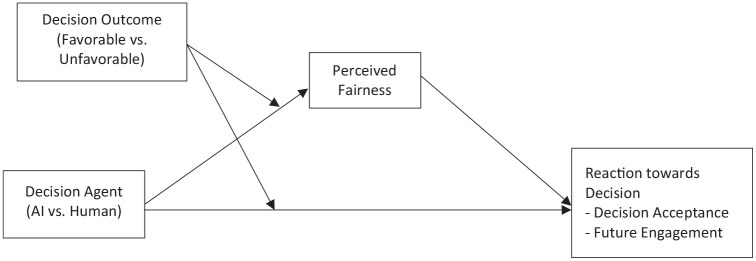
Theoretical Model.

The current research investigates whether decision outcomes (favorable vs. unfavorable to the self) interplay with decision agents (AI vs. human) to shape fairness perception in organizational decision-making contexts and the downstream implication of fairness perception on decision acceptance and future engagement (Figure 1). We posit that when the decision is favorable, individuals have a higher fairness perception regardless of whether the decision was made by AI or human (Hypothesis 1). However, with unfavorable decisions, AI (vs. human) is seen as fairer (Hypothesis 2), leading individuals to accept the decisions and sustain their engagement (Hypothesis 3). Additionally, we examine whether being seen as unemotional, being seen as objective, or a combination of the two influences the formulation of fairness heuristic toward AI (vs. human). Furthermore, we explore whether learning about AI’s capacity to be biased impacts the perceived fairness of AI and subsequent reactions to algorithmic decisions in the face of unfavorable outcome.

## Research Overview

We conducted six experiments to test our hypotheses and to examine the generalizability of the findings across different geographic regions and workplace settings. Study 1 examined whether a decision (unfavorable vs. favorable) leads to differential fairness perception toward AI (vs. human) in the context of an internship conflict among business school students in Asia (Hypotheses 1 and 2). Study 2 investigated the full theoretical model of fairness perception and its downstream consequence in the context of workplace resource allocation (Hypotheses 1, 2, and 3) among working adults in the U.S. Study 3A further tested the full theoretical model toward culpability judgment in workplace (Hypotheses 1, 2, and 3) among American working adults. Study 3B was a pre-registered study that replicated Study 3A with a diverse global online sample to examine the generalizability of the effects across different populations. Study 4 explored the generalizability of the effect to a behavioral outcome using a task performance measure as a proxy for future engagement and explored “unemotional” and “objective” as potential mechanisms shaping perceived fairness. Given the increasing media coverage of AI biases in decision-making, Study 5 examined whether a reminder about AI biases impacts the fairness perception of AI (vs. human) as a decision agent. Studies in this research were approved by the Committee on Research Practices at the researchers’ institution. Studies 1, 2, 3A, and 4 were not pre-registered; the design, sample size, exclusion criteria, and analysis plan for Studies 3B and 5 were pre-registered. The data and materials for all studies and the preregistrations for Studies 3B and 5 are accessible in the Open Science Framework: https://osf.io/h4euc/. We report how we determined our sample size, all data exclusions (if any), all manipulations, and all measures in the studies in the manuscript.

## Study 1

Study 1 established the effect of unfavorable (vs. favorable) decision on perception of fairness toward AI (vs. human) as a decision agent.

### Method

#### Participants

Two hundred forty-three undergraduate students at a university in Asia participated in the study in exchange for course credit. The sample size was determined by the number of students available to participate in the study. We excluded 66 participants who failed to follow instructions (e.g., did not summarize scenario), or failed the manipulation check (e.g., failed to recall the decision agent or the decision in the scenario). The final sample consisted of 177 participants (59.3% female, age: *M* = 19.47, *SD* = 2.79), and 66% of this final sample had internship or part-time work experiences. According to a sensitivity analysis for linear regression using G*Power 3.1, this sample size would detect a medium effect size of Cohen’s *f* = .24 with 80% power. The sample distribution and exclusion across conditions for all studies are available in Supplemental Materials (Tables S1, S5, S9, S12, S26, and S29).

#### Procedure

After consenting to participate in the study, the participants read a scenario in which they were asked to take the role of an intern whose company recently implemented a new conflict resolution process to better manage disputes between employees. The scenario provides general background information about the dispute resolution system of the company. The participants were randomly assigned to one of two Decision Agent Conditions. In the HR Manager (vs. HR Artificial Intelligence) Condition, participants read that,

“Upon receiving a complaint, the case would be passed onto a HR manager who has 10 years of experience in handling personnel issues (vs. input into a HR Artificial Intelligence system that has been trained with 10 years’ worth of personnel data). After reviewing all the existing data on similar incidents in the past, the HR manager (vs. HR AI system) would come up with a final resolution for the conflict. The HR manager (vs. HR AI system) would also take each disputant’s previous behaviors at work and company policies into consideration.”

Next, the participants further read that they had a conflict with another intern and need to engage in dispute resolution. The sex of the person in dispute was matched with that of the participant. The scenario noted that the participant and the other intern disagreed on who should receive credit for a new project and a return offer from the company (adapted from Fu et al. 2007). The participants read that the decision was either favorable or unfavorable to them. That is, participants were assigned to one of the 2 (Decision Agent: HR Manager vs. AI System) × 2 (Decision: Favorable vs. Unfavorable) between-subjects conditions. In the Favorable Condition, the participant received credit for the project and a return offer from the company. In the Unfavorable Condition, the participant did not receive any credit nor a return offer. Then, participants summarized the scenario and completed measures of perceived fairness of the decision agent by indicating the extent to which the agent is “fair,” showed “lack of bias,” and is “impartial” (Conlon & Fasolo, 1990) using seven-point scales anchored from “not at all” to “extremely” (α =.91). As a manipulation check, the participants were asked to recall the decision agent and the decision at the end of the study.^2,3^

### Results

See Supplemental Materials for the means and standard deviations for all measures across all cells (Tables S2, S6, S10, S13, S27, and S30) and the correlations between all measures (Tables S3, S7, S11, S14, S28, and S31) for all studies.

To examine the impact of decision on perceived fairness of AI (vs. human) decision agent, we conducted moderation analyses with PROCESS Model 1 (Hayes, 2018; 5,000 bootstrap samples). In the model, decision agent (0 = HR Manager, 1 = AI System) was entered as an independent variable, decision (0 = Favorable, 1 = Unfavorable) was entered as a moderator, and perceived fairness was entered as a dependent variable.

**Figure 2.**
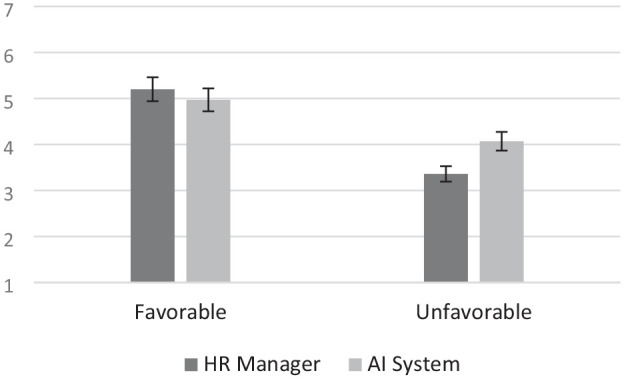
Post-resolution Perception of Fairness toward HR Manager vs. AI System in Study 1. *Note.* Error bars represent 95% confidence interval.

Results revealed a significant main effect of Decision, *B* = −1.84, *SE* = .25, *p* < .001, 95% CI [−2.33, −1.35]; fairness evaluation was higher when the decision was favorable (vs. unfavorable). The main effect of Decision Agent on perceived fairness was not significant, *B* = −.23, *SE* = .25, *p* = .3647, 95% CI [−.73, .27]. There was a significant Decision Agent by Decision interaction, *B* = .95, *SE* = .36, *p* = .0092, 95% CI [.24, 1.66]. Analyses on the conditional effects of Decision Agent on perceived fairness showed that the AI system was perceived as significantly fairer than the HR manager when the decision was unfavorable, *B* = .72, *SE* = .26, *p* = .0056, 95% CI [.22, 1.22]. The AI system and the HR manager did not differ in terms of perceived fairness when the decision was favorable, *B* = −.23, *SE* = .25, *p* = .3647, 95% CI [−.73, .27] (Figure 2).

## Study 2

Results from Study 1 showed that decision affects perceived fairness of AI (vs. human) as a decision agent, supporting the first two predictions. Study 2 examined the effect of decision on the perceived fairness and to test its downstream consequence on the extent to which the decision would be accepted.

### Method

#### Participants

We recruited 454 participants from the U.S. on Amazon Mechanical Turk through the CloudResearch.com platform (Litman et al., 2017). The sample size was determined based on budgetary constraints. We excluded 33 participants who failed to provide an accurate summary of the scenario, or failed the manipulation check, or both. There were 421 participants in the final sample (46.6% female, age: *M* = 38.05, *SD* = 11.04), and 92.9% of the final sample was employed at the time of participation. A sensitivity analysis for linear regression using G*Power 3.1 showed that the sample size of this size would detect a small to medium effect size of Cohen’s *f* = .15 with 80% power.

#### Procedure

Participants were randomly assigned to one of the 2 (Decision Agent: HR Manager vs. AI System) × 2 (Decision: Favorable vs. Unfavorable) between-subjects conditions. They read a workplace conflict scenario that was identical to Study 1 with two exceptions. First, given that the respondents were older, instead of taking up the role of an intern, they took the role of an employee having a conflict with another coworker. Second, the dispute was about receiving a bonus payment, instead of receiving a return offer. In the Favorable Condition, the participants were given a bonus. In the Unfavorable Condition, the participants were not rewarded with any bonus. After reading the scenario, the participants briefly summarized the scenario and indicated the perceived fairness of the decision agent (α = .89) as in Study 1. They were also asked to indicate whether they found the decision to be acceptable or not using a seven-point scale (1 = very unacceptable; 7 = very acceptable). As a manipulation check, the participants were asked to recall the decision agent and the decision.

### Results

#### Perceived Fairness

Consistent with Study 1, we conducted moderation analyses with PROCESS Model 1 (Hayes, 2018; 5,000 bootstrap samples) to test the impact of Decision Agent and Decision on perceived fairness of the decision agents.

The main effect of Decision Agent (0 = HR Manager, 1 = AI System) was not significant, *B* = −.31, *SE* = .20, *p* = .1301, 95% CI [−.70, .09]. The main effect of Decision (0 = Favorable, 1 = Unfavorable), *B* = −2.35, *SE* = .20, *p* < .001, 95% CI [−2.74, −1.95] was significant, with higher fairness perception in the favorable (vs. unfavorable) condition. The Decision Agent × Decision interaction effect was significant, *B* = .84, *SE* = .29, *p* = .0036, 95% CI [.28, 1.40]. Analyses on the conditional effects of Decision Agent on perceived fairness showed that the AI system was perceived as significantly fairer than the HR manager in the Unfavorable Condition, *B* = .53, *SE* = .20, *p* = .0093, 95% CI [.13, .93]. There was no significant difference between the AI system and HR manager in the Favorable Condition, *B* = −.31, *SE* = .20, *p* = .1301, 95% CI [−.70, .09].

#### Downstream Effects

**Figure 3.**
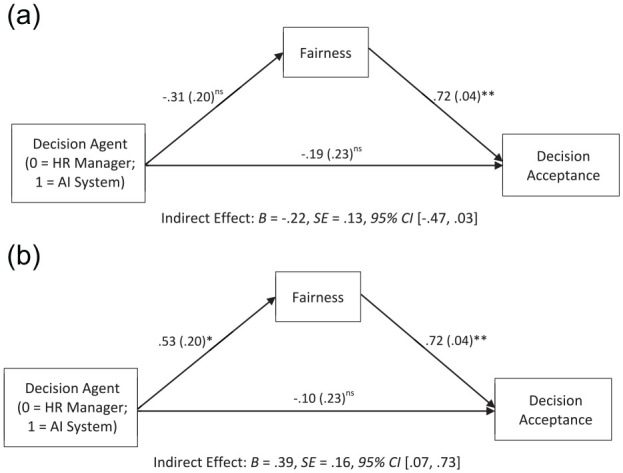
Study 2 Indirect Effect of Decision Agent on Decision Acceptance Through Fairness in (a) Favorable and (b) Unfavorable Outcome Condition ^†^*p* < .10; *p < .05; ** *p* < .*001*.

We tested whether Decision Agent and Decision influenced decision acceptance through fairness perception of the decision agent, using PROCESS Model 8 (Hayes, 2018; 5,000 bootstrap samples). Results indicated that the interaction between the Decision Agent Condition (0 = HR Manager, 1 = AI System) and the Decision Condition (0 = Favorable, 1 = Unfavorable) significantly predicted decision acceptance through perceived fairness of decision agent, *B* = .61, *SE* = .21, 95% CI [.21, 1.03]. Results of conditional indirect effects showed that there was a positive significant indirect effect through fairness in the Unfavorable Condition, but not in the Favorable Condition (Figure 3).

## Studies 3A &3B

Studies 1 and 2 examined the hypothesized effects in the context of conflict resolution. Studies 3A and 3B examined the generalizability of the effects in a different organizational decision-making context, namely culpability judgment. They further examined the downstream impact of decision agents on decision acceptance and future engagement and explored the impact of varying degree of decision favorability. Moreover, Study 3B was a pre-registered study aiming to replicate the effects found in Study 3A with a working adult sample recruited outside the U.S.

### Study 3A Method

#### Participants

Three hundred one participants from the U.S. were recruited on Amazon Mechanical Turk through the CloudResearch.com platform (Litman et al., 2017). The sample size was determined based on budgetary constraints. We excluded 53 participants who failed to provide an accurate summary of the scenario, or failed the manipulation check, or both. The final sample consists of 248 participants (39.5% female, age: *M* = 36.34 years, *SD* = 10.55). At the time of participation, 90.3% of the final sample was employed. According to a sensitivity analysis for linear regression using G*Power 3.1, the sample size of this size would detect a small to medium effect size of Cohen’s *f* = .20 with 80% power.

#### Design and Procedure

After consenting to participate in the study, the participants read a scenario in which they assumed the role of an employee at a transportation company who caused a car accident while speeding on the way to cover a shift for another colleague (adapted from Zemba et al. 2006). The Decision Agent manipulation was introduced the same way as in the previous studies with the following two exceptions. First, based on the scenario, the decision agent (i.e., HR manager or AI system) was involved in determining penalty for the employee who had caused the car accident. In the AI System (vs. HR Manager) Condition, it noted that,

“Your case was input into an Artificial Intelligence system that has 10 years’ worth of personnel data (vs. passed on to a Human Resources manager who has 10 years of experience in handling personnel issues). The AI system (vs. HR manager) would come up with a final decision after reviewing all the existing data on similar incidents in the past. The AI system (vs. HR manager) would also take your previous behaviors at work and company policies into consideration.”

Second, the decisions with varying favorability were adapted to the scenario. The participants were presented with one of three favorability levels: (1) exemption from any penalty (Favorable Condition), (2) suspension from work for three months (Moderate Condition), and (3) immediate termination of employment (Unfavorable Condition). The study involved a 2 (Decision Agent: HR Manager vs. AI System) × 3 (Decision: Favorable vs. Moderate vs. Unfavorable) design. After reading the scenario, they provided a summary of the scenario and evaluated the perceived fairness of the decision agent (α = .84). They indicated their future engagement with the company on a three-item measure (JRA, 2007) using a seven-point scale (1 = strongly disagree; 7 = strongly agree; α = .95) Items were “I feel a sense of commitment to this company,” “Overall, I would recommend this company as a great place to work,” and “I feel inspired to go the extra mile to help this company succeed.”

### Study 3A Results

To test whether the interaction between Decision Agent and Decision predicts decision acceptance and future engagement through perceived fairness of decision agent, we conducted moderated mediation analyses with PROCESS Model 8 (Hayes, 2018; 5,000 bootstrap samples). We used the following coding for Decision (W1: −1 = Favorable, 1 = Moderate, 0 = Unfavorable; W2: −1 = Favorable, 0 = Moderate, 1 = Unfavorable). W1 compares the Favorable Condition to the Moderate Condition, and W2 compares the Favorable Condition to the Unfavorable Condition.

#### Decision Acceptance

**Figure 4.**
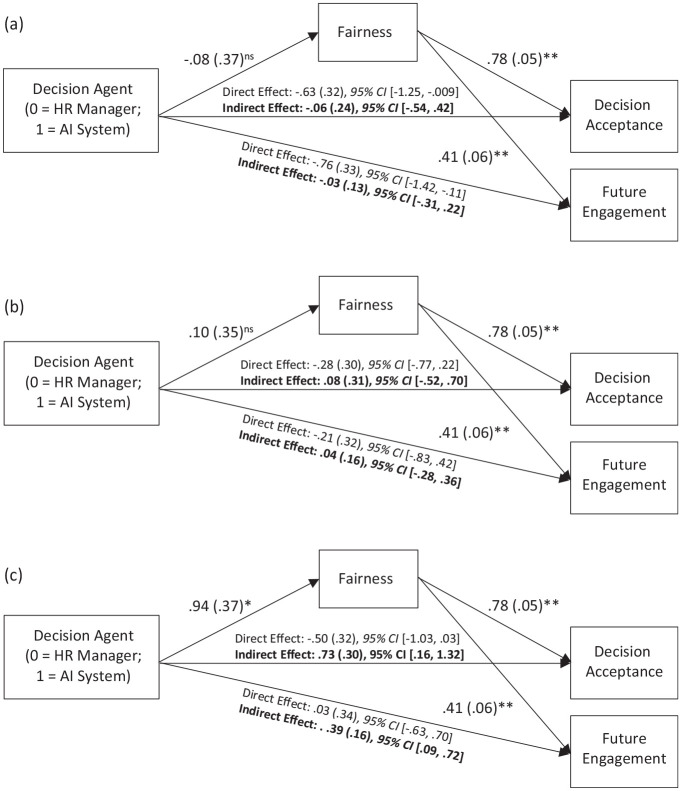
Study 3A Indirect Effect of Decision Agent on Decision Acceptance and Future Engagement Through Fairness in (a) Favorable, (b), Moderate, and (c) Unfavorable Decision Condition ^†^*p* < .10; *p < .05; ** *p* < .*001.*

Results showed that the interaction between Decision Agent Condition (0 = HR Manager, 1 = AI System) and Favorable (vs. Moderate) Condition (W1) did not predict decision acceptance through perceived fairness of the decision agent, *B* = −.17, *SE* = .24, 95% CI [−.65, .31]. However, the interaction between Decision Agent Condition and Favorable (vs. Unfavorable) Decision Condition (W2) significantly predicted decision acceptance through perceived fairness of the decision agent, *B* = .48, *SE* = .24, 95% CI [.03, .96]. Follow-up analyses revealed that the indirect effect through perceived fairness was positive and significant in the Unfavorable Condition, but non-significant in the Favorable and Moderate Conditions (see Figure 4).

#### Future Engagement

The interaction between the Decision Agent Condition (0 = HR Manager, 1 = AI System) and Favorable (vs. Moderate) Decision Condition (W1) did not have a significant effect on future engagement through perceived fairness of the decision agent, *B* = −.09, *SE* = .12, 95% CI [−.33, .16]. However, the interaction between Decision Agent Condition and Favorable (vs. Unfavorable) Decision Condition (W2) significantly predicted future engagement through perceived fairness of the decision agent, *B* = .25, *SE* = .13, 95% CI [.01, .53]. Follow-up analyses showed that the indirect effect through fairness was positive and significant in the Unfavorable Decision Condition, but non-significant in the Favorable and Moderate Decision Conditions (see Figure 4).

The results on the downstream consequences of Decision Agent and Decision interaction through perceived fairness were consistent across Studies 2 and 3A. When the decision was favorable, there was no difference between the attitude toward human vs. algorithmic decision. However, when the decision was unfavorable, the AI system was perceived as fairer than the HR manager, resulting in relatively less negative reaction toward algorithmic decision.

### Study 3B Method

#### Participants

An a priori power analysis based on the results from Study 3A using G*Power 3.1 showed that a sample of 624 was needed to detect the effect of the interaction between decision agents and decision on perceived fairness of decision agent with 90% power. To account for potential exclusions, we recruited 699 participants from the online platform Prolific. To examine the generalizability of the findings, respondents from different global regions other than the U.S. were recruited. We excluded 61 participants who failed to provide an accurate summary of the scenario, or failed the manipulation check, or both. Six hundred thirty-eight participants remained in the final sample (50.9% Europeans, 42% Africans, 3.1% Asians, 2.5% South Americans, .8% North Americans, and .6% Australians; 51.1% female, age: *M* = 30.84 years, *SD* = 9.30). At the time of participation, 94.4% of the final sample was employed and had 7.92 (SD = 8.39) years of work experience on average.

#### Design and Procedure

Study 3B adopted the same design and procedure as in Study 3A. Participants were randomly assigned to one of the conditions in the 2 (Decision Agent: HR Manager vs. AI System) × 3 (Decision: Favorable vs. Medium vs. Unfavorable) design. After reading the scenario about the employee causing a car accident, participants summarized the scenario and evaluated perceived fairness of the decision agent (α = .67).^4^ They also indicated the degree to which they would accept the decision and engage in the company in the scenario (α = .91). They were asked to recall the decision agent and decision in the scenario as a manipulation check.

### Study 3B Results

We used PROCESS Model 8 (Hayes, 2018; 5,000 bootstrap samples) to examine the effect of interaction between Decision Agent and Decision on decision acceptance and future engagement.

#### Decision Acceptance

**Figure 5.**
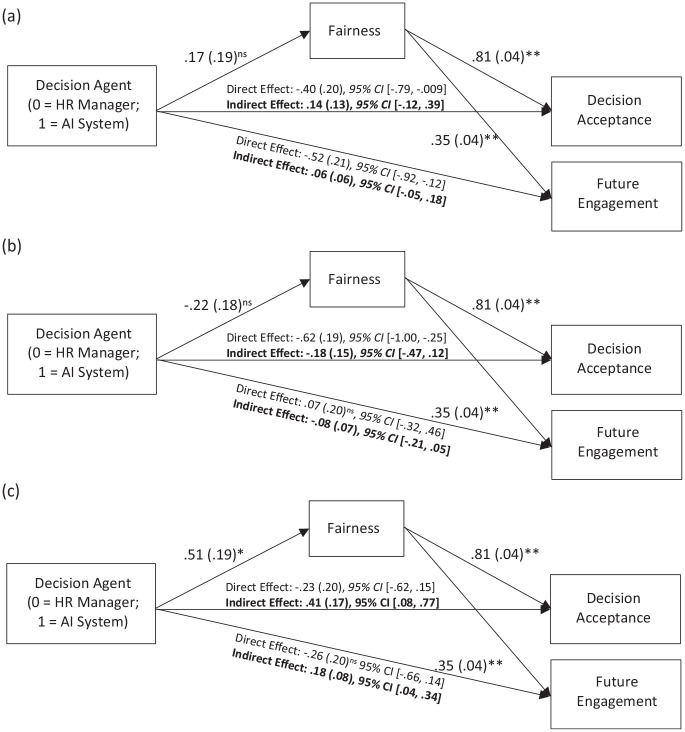
Study 3B Indirect Effect of Decision Agent on Decision Acceptance and Future Engagement Through Fairness in (a) Favorable, (b), Moderate, and (c) Unfavorable Decision Condition †*p* < .10; *p < .05; ** *p* < .*001*.

As expected, the interaction between Decision Agent Condition and Favorable (vs. Unfavorable) Decision Condition significantly predicted decision acceptance through perceived fairness of the decision agent, *B* = .29, *SE* = .13, 95% CI [.03, .54]. The indirect effect of Decision Agent Condition and Favorable (vs. Moderate) Decision Condition interaction on decision acceptance was also significant, B = −.30, SE = .12, 95% CI [−.55, −.06]. This latter effect was unexpected. However, we do not interpret this effect further because follow-up analyses on the conditional indirect effects revealed that the indirect effect through perceived fairness was positive and significant in the Unfavorable Condition only, but non-significant in the Favorable and Moderate Conditions (see Figure 5).

#### Future Engagement

As expected, the interaction between Decision Agent Condition and Favorable (vs. Unfavorable) Decision Condition had significant indirect effects on future engagement through perceived fairness, *B* = .12, *SE* = .06, 95% CI [.02, .24]. The interaction between Decision Agent Condition and Favorable (vs. Moderate) Decision Condition on engagement through fairness perception also was significant, *B* = −.13, *SE* = .06, 95% CI [−.25, −.03]. This latter effect was unexpected. However, we do not interpret this effect further, because follow-up analyses showed that the indirect effect was significant only when the decision was unfavorable, but non-significant when the decision was favorable or moderate (see Figure 5).

Given that Studies 3A and 3B adopted the same design, we conducted additional analyses with the combined sample (N = 886). The results were consistent with those from Studies 3A and 3B. Results are available in the Supplemental Analyses (Tables S25 and S26).

## Study 4

Study 3 showed that higher fairness perception leads to higher intention for future engagement when an unfavorable decision is given by AI (vs. human). Study 4 aimed to explore the potential mechanisms influencing fairness perception. Past research on machine heuristics has shown that technological agents are often perceived as more objective and unemotional compared with humans, and consequently less prone to biases (e.g., Araujo et al., 2020; Jago & Laurin, 2022; Sundar & Nass; 2001). Thus, Study 4 examined whether AI (vs. human) is perceived as more objective and unemotional, and consequently attributed a higher level of fairness in the context of unfavorable decisions. In addition, Study 4 also explored the downstream consequence of fairness perception on future engagement using a behavioral measure. A performance measure was used as a proxy for future engagement based on the principles of fairness heuristic theory. Fairness heuristic theory argues that when individuals perceive an entity as fair, they are more likely to invest personal resources (e.g., time, effort) into their role or task, as they believe that their investment will not be exploited (Lind, 2001; van den Bos et al., 1997). Past studies corroborate this argument, showing that employees who had a higher fairness perception toward organizations exhibited higher levels of performance (Aryee et al., 2004; Johnson et al., 2009). In the context of algorithmic decision-making, higher fairness perception toward an algorithmic decision agent leads to higher productivity of employees (Bai et al., 2022). Moreover, engagement refers to the extent to which individuals are invested in a role or a task and is a crucial determinant of performance (Harter et al., 2002; Rich et al., 2010). Highly engaged employees are more likely to exert greater effort, positively impacting their performance (Vogelgesang et al., 2013). This suggests that the level of engagement following a decision from AI (vs. human) could be reflected in the decision recipients’ task performance.

## Method

### Participants

Two hundred forty-two undergraduate students at a university in Asia participated in the study in exchange for course credit. The sample size was determined by the number of students available to participate in the study. After excluding 24 participants who failed to follow instructions (e.g., fail to complete the task, fail to comply with the task setting), 218 participants remained in the final sample (58.3% female, age: *M* = 20.05 years, *SD* = 6.81), and 76.1% of the final sample had internship or part-time work experiences. A sensitivity analysis for linear regression using G*Power 3.1 showed that the sample size of this size would detect a small to medium effect size of Cohen’s f = .19 with 80% power.

### Procedure

After providing consent to participate, the participants were asked to complete an agility test. The test was to click as many dots as possible in a fixed amount of time when they appear on a screen (Nerd or Die, 2021). The participants were instructed that their performance in the practice round would be assessed by a decision agent that was either an Artificial Intelligence or a Research Assistant. The participants were told that if their performance was deemed satisfactory by the decision agent, they would proceed to the final round immediately. However, if their performance was deemed unsatisfactory, they would be required to complete five additional rounds of practice before they could complete the final round. The need to complete five more rounds would be seen as unfavorable, because this means that the participants would need to spend additional time on the task. Following the practice round, regardless of their performance, all participants received the unfavorable decision that they should complete five additional practices. After completing five additional rounds of practice, they proceeded to the final round. They then completed measures on objectivity and unemotionality of the decision agent on scales anchored at “1 = Not at all” to “7 = Extremely.” Perceived fairness was also measured as in previous studies (α = .82). The final performance score was used as a downstream outcome after controlling for variations in initial performance.

### Results

**Figure 6.**
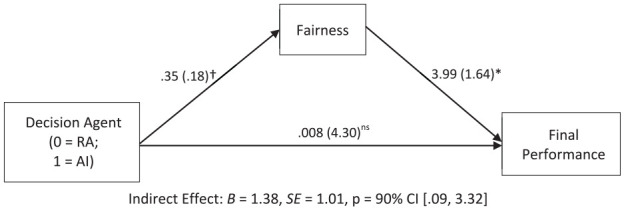
Study 4 Indirect Effect of Decision Agent on Final Performance Through Fairness. ^†^*p* < .10; *p < .05; ** *p* < .*001.*

We used PROCESS Model 4 (Hayes, 2018; 5,000 bootstrap samples) to investigate the indirect effect of Decision Agent on the final performance score through fairness perception. In the model, Decision Agent (0 = Research Assistant, 1 = AI Assistant) was entered as the independent variable, fairness was entered as a mediator, and final performance score was entered as the dependent variable. We also entered the score from the initial round as a covariate to account for the variation in the initial performance. As shown in Figure 6, results revealed that there was an indirect effect of Decision Agent on the final performance through fairness perception; AI was perceived fairer than RA, and such higher fairness perception led to higher final score.

**Figure 7.**
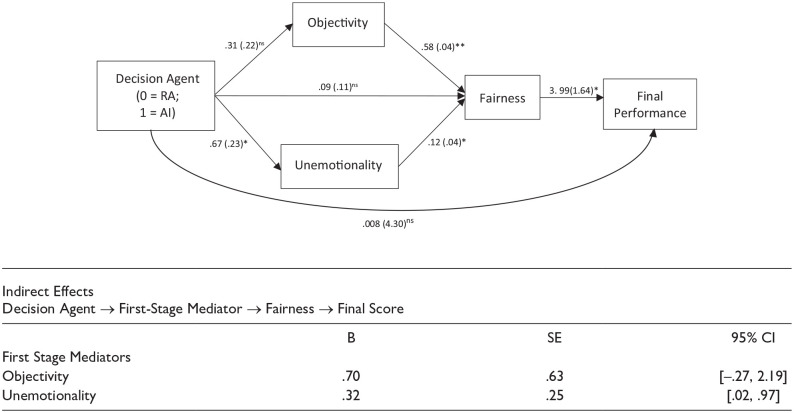
Study 4 Moderated Serial Mediation. ^†^p < .10; *p < .05; ** p < .001.

We then used PROCESS (Hayes, 2018; 5,000 bootstrap samples) to build a serial mediation model with Decision Agent as the independent variable, perceived objectivity and unemotionality as the first-stage mediators, fairness as the second-stage mediator, final performance score as the dependent variable, and the initial performance score as the covariate. As shown in Figure 7, results indicated that Decision Agent Condition (0 = RA, 1 = AI) significantly predicted perceived fairness of decision agent through perceived objectivity and unemotionality of the decision agent. The results also showed that the fairness perception of decision agent explained final performance. There was a significant indirect effect on the final performance through perceived unemotionality as the first-stage mediator and fairness as the second-stage mediator, but not through perceived objectivity as the first-stage mediator. These findings indicate that, in the face of an unfavorable decision, AI assistant is perceived more unemotional, and subsequently perceived fairer than RA. Such higher fairness perception leads to engagement in higher final performance score.

## Study 5

Studies 1–4 provided converging evidence showing that when the decision is unfavorable, decision recipients perceived AI (vs. human) as fairer, showing higher decision acceptance and future engagement. Recently, there has been an increasing discussion on AI biases due to biased human input (Bousquette, 2023; Dastin, 2018; Nicoletti & Bass, 2023). Given the malleability of one’s perception toward AI (Jago & Laurin, 2022) and the dynamic nature of fairness perception (Proudfoot & Lind, 2015), highlighting AI biases might impact the perception of fairness toward AI. Study 5 was a pre-registered experiment that aimed to test whether learning about AI biases influences the perceived fairness of AI, and consequently impacts reactions toward AI decisions in the face of unfavorable outcome.

### Method

#### Participants

An a priori power analysis based on Study 4 results using G*Power 3.1 showed that a sample of 610 was needed to detect the effect of the AI (vs. human) decision agent conditions on perceived fairness with 90% power. To account for having an additional condition that highlighted biases in AI and potential exclusions, we recruited 1,208 global participants from the online platform Prolific.^5^ After excluding 116 participants who failed to provide an accurate summary of the scenario, or failed the manipulation check, or both, 1,092 participants remained in the final sample (57.1% Europeans, 32.8% Africans, 4.7% Asians, 2.2% North Americans, 1.6% South Americans, and 1.6% Oceanians; 50.5% female, age: *M* = 31.44 years, *SD* = 10.21). At the time of participation, 78.2% of the final sample was employed and had 8.34 (SD = 9.48) years of work experience on average.

#### Procedure

Participants read a workplace conflict scenario that was identical to the scenario used in Study 2 except for the following modifications. First, participants were randomly assigned to one of the following three Decision Agent Conditions: (1) HR Manager, (2) AI System, and (3) AI Bias. After reading the conflict, participants in the HR Manager (vs. AI System) Condition read that,

“Your case was passed onto a Human Resources manager (vs. an Artificial Intelligence system) with access to personnel management policies and procedures manuals of the company. The HR manager (vs. HR AI system) would come up with a final decision after reviewing your case based on this information.”

We adjusted the decision agent manipulation from Study 2 to control for the quantity and quality of the data a decision agent has access to, such that the final decision from either the HR Manager or AI System would be based on an identical set of pre-generated information (i.e., the company’s personnel management policies and procedures manuals). Furthermore, in the AI Bias Condition, in addition to reading the information in the AI system condition, the participants also read the following paragraph about how AI might replicate human biases (adapted from Jago & Laurin, 2022).

“You have come across news that AI algorithms learn to replicate human decisions. To train AI, organizations generally provide it with big datasets where it figures out the factors that affected decisions human managers made across different contexts. After figuring out which factors were most associated with certain decisions, this algorithm ‘looks’ for those factors to generate a decision in a new situation. What this means is that the algorithm can actually learn to replicate human biases, if managers tended to make biased decisions.”

Furthermore, as we have already established that individuals form differential fairness perception toward AI (vs. human) in the face of unfavorable decisions, all participants in the current study received an unfavorable decision (i.e., not receiving any bonus). After reading the scenario, the participants briefly summarized the scenario and indicated the perceived fairness of the decision agent (α = .71).^6^ They also indicated the degree to which they would accept the decision and their engagement with the company (α = .90). Participants were also asked to recall the decision agent and the decision as a manipulation check.

### Results

We tested whether Decision Agent Condition influences fairness perception of the decision agent, and the implications of perceived fairness. We conducted mediation analyses using PROCESS Model 4 (Hayes, 2018; 5,000 bootstrap samples). In the model, Decision Agent Condition was entered as an independent variable, fairness as a mediator, and decision acceptance and future engagement as separate dependent variables.

We first conducted analyses contrasting the AI System and AI Bias Conditions to the HR Manager Condition. We used the following coding for Decision Agent (X1: −1 = HR Manager, 1 = AI System, 0 = AI Bias; X2: −1 = HR Manager, 0 = AI System, 1 = AI Bias). X1 compares the HR Manager Condition to the AI System Condition, and X2 compares the HR Manager Condition to the AI Bias Condition. Results showed that, consistent with Studies 1 to 4, HR Manager (vs. AI System) Condition (X1) significantly predicted perceived fairness of the decision agent, *B* = .14, *SE* = .06, *p* = .0208, 95% CI [.02, .26]. Specifically, AI system was perceived as significantly fairer than HR manager. Importantly, HR Manager (vs. AI Bias) Condition (X2) did not significantly predict perceived fairness of the decision agent, *B* = .01, *SE* = .06, *p* = .8616, 95% CI [−.11, .13]. The results demonstrated that when AI biases are highlighted, AI system as a decision agent was not perceived differently from human decision agent in terms of fairness.

As shown in Table 1, replicating the findings in Studies 1–4, the higher fairness perception toward AI System (vs. HR Manager) significantly predicted decision acceptance and future engagement. However, the effect of HR Manager (vs. AI Bias) Condition on decision acceptance and future engagement through perceived fairness was not significant. Taken together, the results suggested that AI is assumed to make fairer decisions than human by default. The halo toward AI decision could be reduced by highlighting potential biases in AI.

**Table 1.** Study 5 Mediation Analyses of Decision Agent on Decision Acceptance and Future Engagement Through Fairness as a Mediator.

	B	SE	95% CI	
			LL	UL
**Decision Agent** → **Fairness** → **Decision Acceptance**				
X1 (HR Manager = −1; AI System = 1)	.10	.04	.01	.18
X2 (HR Manager = −1; AI Bias = 1)	.008	.04	–.08	.09
**Decision Agent** → **Fairness** → **Future Engagement**				
X1 (HR Manager = −1; AI System = 1)	.06	.03	.01	.11
X2 (HR Manager = −1; AI Bias = 1)	.004	.03	–.05	.05

We then conducted analyses contrasting AI Bias and HR Manager Conditions to the AI System Condition. The following coding was used for Decision Agent (X1: −1 = AI System, 1 = AI Bias, 0 = HR Manager; X2: −1 = AI System, 0 = AI Bias, 1 = HR Manager). X1 compares the AI System Condition to the AI Bias Condition, and X2 compares the AI System Condition to the HR Manager Condition. As the results contrasting the AI System Condition and the HR Manager Condition are reported above, we report the results comparing the AI System Condition to the AI Bias Condition. Results showed that AI System (vs. AI Bias) Condition (X1) did not significantly predict perceived fairness of the decision agent, *B* = .01, *SE* = .06, *p* = .8616, 95% CI [−.11, .13]. Naturally, the AI System (vs. AI Bias) Condition did not significantly predict decision acceptance, *B* = .008, *SE* = .04, 95% CI [−.08, .09], or future engagement, *B* = .004, *SE* = .02, 95% CI [−.05, .05], through perceived fairness. This suggests that while highlighting potential biases in AI systems can diminish their perceived fairness, the initial perception of AI as fairer than humans may still persist to some degree. This means that biased AI systems might be perceived as falling somewhere between AI systems without highlighted biases and humans in terms of fairness.

## General Discussion

The increasing use of AI in making high-stake decisions within organizations has raised significant concerns about fairness and reactions to algorithmic decisions (e.g., Gilbert, 2023; Russon, 2020; Solon et al., 2022). The current research investigates how fairness perceptions are shaped by the decision outcomes (unfavorable vs. favorable) and the decision agents (AI vs. human) and examines the downstream implications of these perceptions. Through six experimental studies, we found that (a) perceived fairness was higher when the decision outcome was favorable (vs. unfavorable) regardless of who the decision agent was, (b) AI was viewed as fairer than human when the decision was unfavorable, (c) individuals reacted less negatively towards algorithmic decision through higher fairness perception in the face of unfavorable decision,^7^ (d) the heightened fairness attributed to AI (vs. human) is due to the perception of AI as an unemotional entity, and (e) highlighting AI biases attenuates the differential fairness perception toward AI (vs. human).

### Theoretical Implications

#### Boundary conditions to algorithmic fairness

Existing research has highlighted the importance of understanding fairness perceptions in the context of algorithmic decision-making (Bai et al., 2022; Langer et al., 2019; Lee, 2018; Marcinkowski et al., 2020; Newman et al., 2020). Whereas some studies found that AI decision agents are seen as fairer than human decision agents, others found that human decision agents are seen as fairer than AI. The divergent findings from this body of research highlight the need for more systematic exploration to identify the boundary conditions that influence fairness perceptions toward AI. Drawing on fairness heuristic theory, we posit that individuals formulate a general impression about fairness based on the attributes of the decision agent and situational circumstances. Specifically, our research systematically examined the favorability of decision outcomes, which have been shown to be closely associated with perceptions of fairness in organizations (e.g., Ambrose et al., 1991; Conlon, 1993) and to interact with decision agents to impact fairness perceptions (e.g., Kouchaki et al., 2015). Our findings suggest that perceptions of algorithmic fairness are dynamic. They highlight that, for a holistic understanding of algorithmic fairness, it is critical to recognize the malleability of perception of AI—the sense of fairness can be spontaneously shaped by situational cues—rather than assuming that fairness perceptions toward algorithmic decisions are static.

By examining the impact of decision favorability on organizational members’ perceptions of algorithmic fairness, the current research extends the understanding of how outcome favorability influences reactions to algorithmic decisions. Existing research, such as Yalcin et al. (2022), found that applicants reacted more positively to human (vs. algorithmic) decision makers when the decision was favorable (i.e., acceptance of application), but this preference did not hold when the decision was unfavorable (i.e., rejection of application). Our study found that when decisions were favorable, organizational members did not distinguish between human and algorithmic decision agents in terms of fairness. However, when decisions were unfavorable, they perceived algorithmic decision agents as fairer than humans. Although our findings may initially seem contradictory to the existing research, a closer examination suggests that the current research might have uncovered a previously unknown piece of a larger puzzle about reactions to algorithmic decisions, highlighting the importance of understanding subtle contextual influences. In Yalcin et al. (2022), the decision recipients were focused on building new relationships with organizations—such as becoming a member or gaining new resources—making the situations gain-oriented. In this context, a favorable decision determined whether respondents would gain new relationships or opportunities, while an unfavorable decision meant they would not. Conversely, in our study, respondents already had ongoing relationships with their organizations, with established histories of past commitments. These situations were loss-oriented, where a favorable decision determined whether respondents would retain their existing relationships, standing, and resources, while an unfavorable decision indicated a potential loss. Taken together, the findings suggest that when the situation is gain-focused, individuals are more likely to differentiate between algorithmic and human decisions in the face of positive outcomes, whereas in loss-oriented situations, individuals tend to react more discriminately to negative outcomes. This highlights the need for future research to go beyond simply asking whether people react positively or negatively to algorithmic decisions. Instead, researchers should consider how the alignment between decision contexts and situational goals interacts with decision agents and outcomes, potentially influencing individual reactions and their implications for personal goal pursuit and engagement (Brockner & Higgins, 2001; Higgins, 2005).

Altogether, despite the considerable research into how people react to algorithmic decisions, much remains unknown and ripe for further exploration. Rather than viewing any single research outcome as definitive, especially as algorithmic decision-making becomes more prevalent across various organizational settings and as people’s understanding of these processes evolves, we can anticipate divergent research findings that will require deeper theoretical and empirical integration.

#### Human vs. AI Differentiation

Research on computer–human interaction presents two contrasting arguments on how individuals perceive and interact with AI agents. One line of work argues that, when interacting with computational systems, individuals would apply social principles that are similar to the ones they apply in their interaction with other humans (Reeves & Nass, 1996). Another line of research argues that people would distinguish the ways they interact with AI agents from humans because AI is seen as a system that lacks human qualities (Gray & Wegner, 2012; Waytz & Norton, 2014). By examining the role of decision agents and decision favorability in shaping individuals’ fairness perception, the current research increases our understanding of the circumstances under which individuals may (or may not) differentiate their attitudes toward AI versus human decision agents. Specifically, when the decision is favorable, people base their assessment of fairness on the decision outcome and do not show differential reactions toward AI and human decision agents, providing support for the view that individuals interact with AI as they would with humans. However, when the decision is unfavorable, individuals are more likely to assess AI based on the qualities that make it distinct from humans, which consequently leads to differential reactions toward decisions made by AI (vs. human). This supports the perspective that individuals interact with AI and human decision agents in a different manner. Taken together, the current research paves the way for future investigation to explore when and why algorithmic agents would be treated similarly or differently compared with traditional human agents.

#### Algorithmic discrimination

Importantly, findings from the current research also enhance our understanding of an emerging line of research on algorithmic discrimination, which examines the perception of an algorithm’s capacity to reinforce discriminatory practices and how individuals respond to such discrimination when it happens. People generally perceive algorithms as less likely to engage in discrimination than humans (Jago & Laurin, 2022) and are less outraged by algorithms (vs. humans) if discrimination occurs (Bigman et al., 2023). The current study further adds to this line of research by enhancing the understanding of how and why people who are subjected to algorithmic discrimination might react. Existing studies on algorithmic discrimination examined the perception of algorithmic discrimination based on anticipated discrimination by algorithms (Jago & Laurin, 2022), or having individuals evaluate algorithmic discrimination from a third-person perspective (Bigman et al., 2023). Our study examined how individuals react to algorithmic (vs. human) decisions that are unfavorable to the selves, which might or might not be attributable to discrimination. Our results show that, in the face of unfavorable decisions, individuals are more likely to make sense of such decisions from AI (vs. human) by attributing higher fairness to AI (vs. human), thus reacting less negatively to such decisions. In other words, the unfavorable decisions from AI (vs. human) are seen to be based on a more solid ground and thus better justified, as it is seen as lacking bias and more impartial than humans. These findings suggest that, in the face of discrimination, individuals may readily accept a decision that disadvantages them if it comes from AI (vs. human). Moreover, in line with the previous research showing that AI is perceived as less emotional than humans and is less likely to be an agent of discrimination (Jago & Laurin, 2022), our study provides evidence that the perception of AI as an unemotional entity contributes to the higher perceived fairness of AI (vs. human). Importantly, when individuals are reminded of biases in AI, the fairness halo of AI over humans as decision agents dissipates. This suggests that one approach to address the potential concerns about algorithmic discrimination is to provide a more balanced discussion about the benefits, as well as the limitations and potential biases, of algorithmic decisions.

#### AI anthropomorphism

Lastly, this research adds to the literature on AI anthropomorphism. Previous studies on anthropomorphism have shown that attributing human-like qualities to non-human entities can help people apply human social norms and expectations in their interactions with these entities (Zlotowski et al., 2015). Most of the research in this area has focused on the positive effects of anthropomorphism on computer-human interaction, demonstrating that imparting AI with human characteristics such as emotions, gender, and moral stances can increase likeability (Roesler et al., 2021; Salem et al., 2013), trust (Plaks et al., 2022; Waytz et al., 2014 ), and acceptance of the computer agents (Cornelius & Leidner, 2021; Eyssel et al., 2012). Our research expands this line of research by showing that attributing human-like qualities to AI agents, like judgment biases, can lead individuals to judge AI and human more similarly, neutralizing the fairness halo given to AI. Specifically, findings from Study 5 showed that informing people about AI’s capacity to replicate human biases diminishes people’s favoritism toward AI (vs. human) in terms of fairness and positive reaction toward algorithmic (vs. human) decisions. These results suggest that making algorithmic agents more human-like may remove characteristics that make these agents superior to humans, leading to potentially positive and negative consequences. On the one hand, equating AI to humans could protect decision recipients from blindly accepting disadvantageous decisions with a false sense of fairness. On the other hand, anthropomorphizing AI agents may be detrimental to organizations by undermining decision recipients’ perceived legitimacy of the organizational decisions made by AI. Based on our findings, future research may further examine the potential upside and downside of anthropomorphizing AI agents by investigating when the humanization of AI agents may enhance, neutralize, or hinder human–AI interaction.

### Practical Implications

This research highlights the need for organizations and their members to be more mindful of the default tendency to view AI decisions as inherently fairer than those made by humans. Organizations and their stakeholders must actively work toward minimizing potential biases in algorithmic decision-making. Despite the prevailing perception of AI as a fair entity, there have been increasing concerns that algorithmic decisions can be biased when it is based on biased human input (Manyika et al., 2019; Nouri, 2021). This means that, in some cases, a false sense of fairness may give legitimacy to algorithmic decisions and propagate existing social inequalities (Rainie & Anderson, 2017), especially when individuals readily accept decisions made by AI. However, the good news is that, as shown in Study 5, increasing people’s awareness about AI biases can attenuate the fairness halo attributed to AI over humans. This signifies that people’s initial belief in AI’s inherent fairness is not deeply ingrained in their mindsets. Thus, raising awareness about AI’s potential biases can change people’s perceptions, suggesting that training and education are effective tools for improving AI literacy. Taken together, the current research provides important implications to both organizations and individuals. It is crucial for organizations to carefully assess the impacts of potential biases in their AI-based decision-making processes and to help employees improve their understanding of AI to prevent their employees from falling prey to the illusion of fairness. Moreover, it is crucial for employees and the public to continuously educate themselves on how AI functions and makes decisions to be able to identify and raise concerns about potential issues in the algorithmic decision-making processes.

What specific actions can individuals and organizations take to increase awareness of AI’s capacity to be biased and address the potential biases embedded in algorithmic decisions? We suggest that, first, organizations can provide workshops to inform employees about AI-based decision-making processes. Although companies are increasingly offering AI-related courses and workshops, these often focus on the technical aspects about how employees can implement or use AI in their work, rather than on potential biases of AI in decision-making or how AI influences the recipients of its decisions (Bratton, 2024; Liu, 2024). By offering workshops that provide a balanced view about the use of AI, its implementation, and its potential biases and limitations, organizations can educate their employees about the strengths and weaknesses of AI-based decisions and break the silent default assumption that AI must be an impartial tool that helps to make fair decisions. At the same time, employees, as individuals, should also keep themselves up to date on topics such as the ethics of AI, addressing issues such as the challenges associated with AI decision-making (e.g., how AI might create inequalities) and the importance of data quality (IBM, 2024; LSE, 2024). By participating in such programs, employees can critically assess and update their assumptions about AI, ensuring they are well-informed and are aware of the latest developments.

Second, companies can conduct regular AI audits to detect any biases or flaws in their AI-based decision-making systems. There is growing recognition of the importance of AI audits, which involve evaluating the training data, algorithms, and results produced by AI systems to ensure they are trustworthy (Somani, 2024). Companies can conduct internal audits of their AI systems on various criteria, such as how well the training datasets represent different groups of employees, and how well their decisions comply with industry regulations or exemplary practices in the industry. Additionally, companies can use services from external firms that provide real-time feedback on potential issues with their AI models, such as data privacy concerns (Credo AI, 2024). Given the increasing emphasis on diversity, equity, and inclusion (DEI) initiatives in organizations, companies may also consider examining the impact of the use of AI on DEI as part of their audit process. Sharing the results of these audits with employees can keep them informed about the performance and ethical standing of the AI systems in use. For example, Microsoft recently published its first annual Responsible AI Transparency Report, which provides insights into their practices for developing, deploying, and managing AI systems responsibly (Microsoft, 2024). Similarly, companies can publish reports disclosing the evaluations of their AI systems, based on audit findings, to share with both their employees and the public. This approach can foster a culture of transparency and accountability in algorithmic decision-making.

### Limitations and Future Directions

Despite the contributions of the current study, it also has some limitations. First, even though our study investigated individuals’ reactions towards algorithmic (vs. human) decisions in the contexts of resolving workplace conflicts, allocating resources, implementing punishment due to rule violation, and evaluating performance, it would be important to test the generalizability of the findings in other contexts. For instance, it would be worthwhile to test whether the current findings can be replicated in different organizational decision domains (e.g., hiring, promotion) and other social contexts (e.g., conflict mediation, family disputes). Moreover, as noted above, future studies can examine whether and how the fit between “gain” versus “loss” decision contexts might interplay with the decision agents and decision outcomes to influence personal goal pursuit and engagement (Brockner & Higgins, 2001; Higgins, 2005).

Second, with the increasing efforts to make AI more “human-like,” the fairness perception toward AI may be reconstructed. In this line of inquiry, future research can examine factors that may contribute to the reconstruction of fairness perception in AI–human interaction. According to fairness heuristic theory, individuals re-evaluate previously formed fairness heuristics when their expectations of a decision authority are violated, or when their relationships with the decision agent go through significant changes (Proudfoot & Lind, 2015). Based on this contention and our finding that the perception of AI as an unemotional entity leads to higher perceived fairness, future research may examine whether adding emotional features to AI impacts the perceived fairness of AI. Recently, there have been increasing efforts to build an AI with the ability to provide responses that convey empathy and emotional support (Mahdawi, 2023). Arguably, AI’s emotional capabilities may violate individuals’ expectations of AI as an unemotional entity, which may result in the reconstruction of the fairness perception associated with AI. For example, even though unfavorable decisions are often received more positively when communicated with empathy (Bies, 2013), unfavorable decisions made by AI with empathy might harm the perception of fairness, leading to more negative reactions.

In a related vein, future research may further explore the conditions under which the positive impact of a decision agent’s unemotionality on fairness perceptions might be diminished. Existing research on organizational justice demonstrates that the interpersonal treatment individuals receive when organizational decisions are made and communicated significantly impacts their perception of fairness (Bies, 2001; Colquitt, 2001). Specifically, treating decision recipients politely and respectfully positively influences decision recipients’ perception of fairness. Therefore, decision agents’ interpersonal sensitivity—their ability to accurately perceive, understand, and respond to others’ emotions and states (Carney & Harrigan, 2003)—is crucial (Bies, 2013). The current research focused on scenarios where decision agents make decisions based on available information without needing to gather input from the decision recipients. In these contexts, processing information without emotional biases understandably contributes to perceptions of fairness. However, in situations where AI must actively communicate with individuals to facilitate the decision-making process, perceived unemotionality may not necessarily enhance fairness perceptions. For example, there has been increasing attention to implementing AI technology in mediation (Hilborne, 2019; Pietromarchi, 2024). In mediation, the mediator’s ability to understand and cater to the disputants’ emotions is crucial to facilitate the process (Kelly & Kaminskienė, 2016). This means that, in contexts where organizational mediators engage in mediation to resolve disputes between employees, the agents’ emotional capacity can be important. Consequently, future research may examine whether the perceived unemotionality of AI plays a different role in shaping perceptions of fairness in decision-making contexts that require interaction with decision recipients.

Additionally, future research may explore the role of emotionality in communicating decisions. The studies conducted in the current research primarily focused on the decision-making process itself, rather than on how decisions were communicated post-decision. Given the positive impact of delivering decisions in a respectful manner on perceived fairness (Bies, 2013), could AI’s perceived unemotionality, which enhances fairness perception during decision-making processes, potentially harm fairness perception during the communication of those decisions? The lack of emotional engagement during the communication phase might lead to perceptions of decisions as impersonal or insensitive, negatively impacting employees’ perceptions of interpersonal treatment. Examining this potential shift in perception could provide valuable insights into the nuanced role of unemotionality in the context of algorithmic decisions.

Lastly, although the current study compared AI and humans as decision agents, it would be interesting to examine how individuals would react when unfavorable decisions are made jointly by AI and humans. Sometimes, the implementation of AI in decision-making processes takes a hybrid form, in which human a decision agent would incorporate AI recommendations into the final decision (Meissner & Keding, 2021). Future studies may examine whether such a hybrid form is more advantageous in fairness perception than the automation of the entire decision-making process through AI by adding a “human touch” to the decisions or whether it is considered less fair by impairing the impartial image of AI.

## Conclusion

Nowadays, AI is often involved in making decisions that were once made by humans, such as who to hire, promote, or even fire. Such an increasing role of AI in decision-making comes with a growing interest in understanding how individuals would react to algorithmic decisions. The current research reveals a dynamic process through which individuals formulate fairness heuristics toward AI versus human as decision agent. Specifically, when outcomes are favorable, the nature of the decision agent matters less. Importantly, individuals would accept unfavorable decisions more readily when the decisions were made by the presumably fair AI than if the same decisions were made by human. This study sheds light on the impact of emerging technology and decision context on people’s perceptions and attitudes.

## Supplemental Material

sj-docx-1-psp-10.1177_01461672241288338 – Supplemental material for For Me or Against Me? Reactions to AI (vs. Human) Decisions That Are Favorable or Unfavorable to the Self and the Role of Fairness Perception Supplemental material, sj-docx-1-psp-10.1177_01461672241288338 for For Me or Against Me? Reactions to AI (vs. Human) Decisions That Are Favorable or Unfavorable to the Self and the Role of Fairness Perception by Jungmin Choi and Melody M. Chao in Personality and Social Psychology Bulletin

## Notes

1. The branch of AI described in the current definition is machine learning. In addition to machine learning, there are other branches of AI, such as expert systems, robotics, and vision. In the current paper, we define AI this way as (1) the AI technologies that are currently used at organizations are primarily based on machine learning (Google, 2023b), and (2) businesses often use AI and machine learning interchangeably (Google, 2023a).

2. To test whether AI and humans are generally viewed differently in terms of fairness, we also measured participants’ perception of fairness toward the decision agent before they learned about the decision. Results are presented in the Supplemental Materials (p. 6).

3. To rule out the possibility that familiarity with AI decision-making might affect perception toward the decision agent, participants also indicated the extent to which they are familiar with how AIs make decisions (Jago, 2019) in Studies 1, 2, 3B, and 5. All reported effects held after controlling for familiarity with AI decision-making. Results are available in Supplemental Analyses (Table S4, S8, S16, S17, and S32).

4. In addition to investigating fairness perception as a core mechanism, we also explore the potential mediating roles of sociability, morality, and competence as they are among the most commonly assessed dimensions of social perceptions (Leach et al. 2007, Heflick et al. 2011), yet little is known about their roles in the context of algorithmic decision-making. As this study focuses on fairness perception, the results related to sociability, morality, and competence are only reported in the Supplementary Analyses (Tables S15 and S18–S23).

5. In the pre-registration, we recorded 800 as the target sample size due to an administrative error. The initial target of 800 participants was an estimate for the two conditions (AI and human), based on an a priori analysis from Study 4. To account for potential exclusions, we aimed to recruit 400 participants per condition. With the additional condition in Study 5, our recruitment goal was set at 1200 participants (400 per condition, including a buffer for exclusions). The pre-registration mistakenly recorded 800 as the target sample size, covering the two initial conditions.

6. As an extension of Study 3B, we explored whether learning about AI biases impacts people’s perception of sociability, morality, and competence towards AI. Results are reported in the Supplementary Analyses (p. 43).

7. Our supplemental study showed that fairness perception also leads to less organizational deviance when an unfavorable decision is made by AI (vs. human). As organizational deviance is not within the scope of the current paper, the results are reported in Supplemental Materials (p. 45).
