# Supplementary material for: For Me or Against Me? Reactions to AI (vs. Human) Decisions That Are Favorable or Unfavorable to the Self and the Role of Fairness Perception
Source: Pers Soc Psychol Bull. 2024 Oct 24;52(3):671–91. doi: 10.1177/01461672241288338 (PMC12804407; doi:10.1177/01461672241288338)
Supplement: sj-docx-1-psp-10.1177_01461672241288338 – Supplemental material for For Me or Against Me? Reactions to AI (vs. Human) Decisions That Are Favorable or Unfavorable to the Self and the Role of Fairness Perception [file sj-docx-1-psp-10.1177_01461672241288338.docx]

**Supplemental Materials for**

For me or against me?: Reactions to AI (vs. Human) decisions that are favorable or unfavorable to the self

Table of Contents

[Study 1 3](#_Toc159162160)

[**Table S1** Study 1 Sample Distribution across Conditions 3](#_Toc159162161)

[**Table S2** Study 1 Means and Standard Deviations for All Measures across All Cells 4](#_Toc159162162)

[**Table S3** Study 1 Correlations between Variables 5](#_Toc159162163)

[**Perception of fairness toward HR Manager vs. AI System** 6](#_Toc159162164)

[**Table S4** Perception of fairness toward HR Manager vs. AI System, Controlling for Familiarity with AI Decision Making in Study 1 7](#_Toc159162165)

[Study 2 8](#_Toc159162166)

[**Table S5** Study 2 Sample Distribution across Conditions 8](#_Toc159162167)

[**Table S6** Study 2 Means and Standard Deviations for All Measures across All Cells 9](#_Toc159162168)

[**Table S7** Study 2 Correlations between Variables 10](#_Toc159162169)

[**Table S8** Study 2 Moderated Mediation Analyses of Decision Agent by Decision on Decision Acceptance through Fairness as a Mediator, Controlling for Familiarity with AI Decision Making 11](#_Toc159162170)

[Study 3A 12](#_Toc159162171)

[**Table S9** Study 3A Sample Distribution across Conditions 12](#_Toc159162172)

[**Table S10** Study 3A Means and Standard Deviations for All Measures across All Cells 13](#_Toc159162173)

[**Table S11** Study 3A Correlations between Variables 14](#_Toc159162174)

[Study 3B 15](#_Toc159162175)

[**Table S12** Study 3B Sample Distribution across Conditions 15](#_Toc159162176)

[**Table S13** Study 3B Means and Standard Deviations for All Measures across All Cells 16](#_Toc159162177)

[**Table S14** Study 3B Correlations between Variables 18](#_Toc159162178)

[**Table S15** Study 3B Moderation Analyses of Decision Agent by Decision on Perceptions of Sociability, Morality, and Competence toward HR Manager vs. AI System 19](#_Toc159162179)

[**Table S16** Study 3B Moderated Mediation Analyses of Decision Agent by Decision on Decision Acceptance through Fairness as a Mediator, Controlling for Familiarity with AI Decision Making 20](#_Toc159162180)

[**Table S17** Study 3B Moderated Mediation Analyses of Decision Agent by Decision on Future Engagement through Fairness as a Mediator, Controlling for Familiarity with AI Decision Making 21](#_Toc159162181)

[**Table S18** Study 3B Moderated Mediation Analyses of Decision Agent by Decision on Decision Acceptance through Fairness and Sociability as Parallel Mediators 22](#_Toc159162182)

[**Table S19** Study 3B Moderated Mediation Analyses of Decision Agent by Decision on Decision Acceptance through Fairness and Morality as Parallel Mediators 24](#_Toc159162183)

[**Table S20** Study 3B Moderated Mediation Analyses of Decision Agent by Decision on Decision Acceptance through Fairness and Competence as Parallel Mediators 26](#_Toc159162184)

[**Table S21** Study 3B Moderated Mediation Analyses of Decision Agent by Decision on Future Engagement through Fairness and Sociability as Parallel Mediators 28](#_Toc159162185)

[**Table S22** Study 3B Moderated Mediation Analyses of Decision Agent by Decision on Future Engagement through Fairness and Morality as Parallel Mediators 30](#_Toc159162186)

[**Table S23** Study 3B Moderated Mediation Analyses of Decision Agent by Decision on Future Engagement through Fairness and Competence as Parallel Mediators 32](#_Toc159162187)

[**Table S24** Studies 3A and 3B Combined Moderated Mediation Analyses of Decision Agent by Decision on Decision Acceptance through Fairness as a Mediator 34](#_Toc159162188)

[**Table S25** Studies 3A and 3B Moderated Mediation Analyses of Decision Agent by Decision on Future Engagement through Fairness as a Mediator 35](#_Toc159162189)

[Study 4 35](#_Toc159162190)

[**Table S26** Study 4 Sample Distribution across Conditions 36](#_Toc159162191)

[**Table S27** Study 4 Means and Standard Deviations for All Measures across All Cells 37](#_Toc159162192)

[**Table S28** Study 4 Correlations between Variables 38](#_Toc159162193)

[Study 5 39](#_Toc159162194)

[**Table S29** Study 5 Sample Distribution across Conditions 39](#_Toc159162195)

[**Table S30** Study 5 Means and Standard Deviations for All Measures across All Cells 40](#_Toc159162196)

[**Table S31** Study 5 Correlations between Variables 41](#_Toc159162197)

[**Table S32** Study 5 Mediation Analyses of Decision Agent on Decision Acceptance and Future Engagement through Fairness as a Mediator, Controlling for Familiarity with AI Decision Making 42](#_Toc159162198)

[**Study 5 Results on Sociability, Morality, and Competence** 43](#_Toc159162199)

[Supplemental Study 44](#_Toc159162200)

[**Method** 45](#_Toc159162201)

[**Results** 45](#_Toc159162202)

## **Study 1**

### **Table S1** *Study 1 Sample Distribution across Conditions*

| Number of Participants | Conditions | HR AI System | HR Manager |
| --- | --- | --- | --- |
| Initial *n* | Favorable | 60 | 64 |
|  | Unfavorable | 60 | 59 |
| Failed to follow instructions, failed the manipulation check, or both | Favorable | 21 | 13 |
|  | Unfavorable | 15 | 17 |
| Final *n* | Favorable | 39 | 51 |
|  | Unfavorable | 45 | 42 |

### **Table S2** *Study 1 Means and Standard Deviations for All Measures across All Cells*

|  | Decision Agent | Decision | Mean | Standard Deviation | |
| --- | --- | --- | --- | --- | --- |
| General Perception of Fairness | HR Manager |  | 4.27 | | 1.10 |
|  | AI System |  | 5.08 | | 1.17 |
| Fairness After Decision | HR Manager | Favorable | 5.20 | | 1.04 |
|  |  | Unfavorable | 3.36 | | 1.24 |
|  | AI System | Favorable | 4.97 | | 1.26 |
|  |  | Unfavorable | 4.07 | | 1.24 |
| Familiarity with AI Decision Making | HR Manager | Favorable | 3.80 | | 1.40 |
|  |  | Unfavorable | 3.81 | | 1.49 |
|  | AI System | Favorable | 3.00 | | 1.30 |
|  |  | Unfavorable | 3.20 | | 1.46 |

### **Table S3** *Study 1 Correlations between Variables*

| **Variable** | **1** | **2** |
| --- | --- | --- |
| 1. General Perception of Fairness |  |  |
| 2. Fairness After Decision | .45** |  |
| 3. Familiarity with AI Decision Making | -.07 | .07 |

^†^ *p* < .10; ** p < .05; ** p < .01*

### **Perception of fairness toward HR Manager vs. AI System**

To test whether AI and humans are generally viewed differently in terms of fairness, we also measured participants’ perception of fairness towards the decision agent before they learned about the decision. Specifically, right after reading that their company implemented a new conflict resolution process that involves a HR manager (vs. HR AI system), participants rated the extent to which they think the decision agent is fair, using the same three-item fairness measure used across all studies in the paper (α = .85). Simple linear regression was used to examine the effect of Decision Agent Condition (0 = HR Manager, 1 = AI System) on perceived fairness. The results revealed that Decision Agent Condition significantly predicted perceived fairness of the decision agent, R^2^ = .12, *F*(1, 175) = 23.05, *p* < .001; people hold a higher fairness perception towards AI system (vs. HR manager) as a decision agent.

Given the nature of the repeated-measures design, there is a possibility that participants’ general perception of the decision agent, which was measured first, influenced participants’ perception towards the decision agent after receiving the decision. We address this limitation in Study 2 by measuring fairness only after participants receive a decision (Favorable vs. Unfavorable) from a decision agent (HR Manager vs. AI System). Moreover, as the other studies in the paper consistently showed that AI (vs. human) was perceived fairer in the face of unfavorable decision, the findings from Study 1 provide initial evidence that individuals hold differential fairness perception towards AI (vs. human) decision agent when the decision is unfavorable.

### **Table S4** *Perception of fairness toward HR Manager vs. AI System, Controlling for Familiarity with AI Decision Making in Study 1*

| Dependent Variable: Fairness | B | SE | p | 95% CI |
| --- | --- | --- | --- | --- |
| Decision Agent (0 = HR Manager; 1 = AI System) | -.15 | .26 | .5529 | [-.66, .36] |
| Decision (0 = Favorable; 1 = Unfavorable) | -1.84 | .25 | .0000 | [-2.33, -1.35] |
| Decision Agent $\times$ Decision | .93 | .36 | .0105 | [.22, 1.64] |
| Favorable Decision | -.15 | .26 | .5529 | [-.66, .36] |
| Unfavorable Decision | .78 | .26 | .0030 | [.27, 1.28] |
| Familiarity with AI Decision Making | .10 | .06 | .1348 | [-.03, .22] |

## **Study 2**

### **Table S5** *Study 2 Sample Distribution across Conditions*

| Number of Participants | Conditions | AI System | HR Manager |
| --- | --- | --- | --- |
| Initial *n* | Favorable | 119 | 110 |
|  | Unfavorable | 107 | 118 |
| Failed to provide accurate summary of the scenario, failed the manipulation check, or both | Favorable | 10 | 6 |
|  | Unfavorable | 8 | 9 |
| Final *n* | Favorable | 109 | 104 |
|  | Unfavorable | 99 | 109 |

### **Table S6** *Study 2 Means and Standard Deviations for All Measures across All Cells*

|  | Decision Agent | Decision | Mean | Standard Deviation | |
| --- | --- | --- | --- | --- | --- |
| Fairness | HR Manager | Favorable | 5.66 | | 1.21 |
|  |  | Unfavorable | 3.31 | | 1.68 |
|  | AI System | Favorable | 5.35 | | 1.40 |
|  |  | Unfavorable | 3.85 | | 1.55 |
| Decision Acceptance | HR Manager | Favorable | 5.49 | | 1.40 |
|  |  | Unfavorable | 3.02 | | 1.82 |
|  | AI System | Favorable | 5.29 | | 1.52 |
|  |  | Unfavorable | 2.92 | | 1.84 |
| Familiarity with AI Decision Making | HR Manager | Favorable | 4.54 | | 1.23 |
|  |  | Unfavorable | 4.57 | | 1.42 |
|  | AI System | Favorable | 4.45 | | 1.36 |
|  |  | Unfavorable | 4.30 | | 1.46 |

### **Table S7** *Study 2 Correlations between Variables*

| **Variable** | **1** | **2** |
| --- | --- | --- |
| 1. Fairness |  |  |
| 2. Decision Acceptance | .75** |  |
| 3. Familiarity with AI Decision Making | .02 | .06 |

^†^ *p* < .10; ** p < .05; ** p < .01*

### **Table S8** *Study 2 Moderated Mediation Analyses of Decision Agent by Decision on Decision Acceptance through Fairness as a Mediator, Controlling for Familiarity with AI Decision Making*

|  | Direct Effects on Fairness | | | | |  |
| --- | --- | --- | --- | --- | --- | --- |
|  | B | SE | | | 95% CI | |
| Decision Agent (0 = HR Manager; 1 = AI System) | -.30 | .20 | | | [-.70, .09] | |
| Decision (0 = Favorable; 1 = Unfavorable) | -2.35*** | .20 | | | [-2.75, -1.95] | |
| Decision Agent $\times$ Decision | .84** | .29 | | | [.28, 1.41] | |
| Familiarity with AI Decision Making | .02 | .05 | | | [-.08, .12] | |
|  | Direct Effects on Decision Acceptance | | | | |  |
|  | B | | SE | 95% CI | |  |
| Decision Agent | .03 | | .17 | [-.32, .37] | |  |
| Decision | -.78*** | | .20 | [-1.17, -.39] | |  |
| Decision Agent $\times$ Decision | -.50* | | .25 | [-.99, -.005] | |  |
| Familiarity with AI Decision Making | .05 | | .05 | [-.06, .12] | |  |
| Fairness | .72*** | | .04 | [.64, .80] | |  |
|  | Indirect Effects on Decision Acceptance | | | | |  |
|  | B | | SE | 95% CI | |  |
| Indirect Effect through Fairness | .61 | | .21 | [.19, 1.01] | |  |
| Favorable Decision | -.22 | | .13 | [-.47, .03] | |  |
| Unfavorable Decision | .39 | | .17 | [.06, .71] | |  |

*Note.* ^†^p < .10; *p<.05; **p<.01; ***p<.001

## **Study 3A**

### **Table S9** *Study 3A Sample Distribution across Conditions*

| Number of Participants | Conditions | AI System | HR Manager |
| --- | --- | --- | --- |
| Initial *n* | Favorable | 55 | 45 |
|  | Moderate | 46 | 54 |
|  | Unfavorable | 54 | 47 |
| Failed to provide accurate summary of the scenario, failed the manipulation check, or both | Favorable | 13 | 8 |
|  | Moderate | 7 | 4 |
|  | Unfavorable | 8 | 13 |
| Final *n* | Favorable | 42 | 37 |
|  | Moderate | 39 | 50 |
|  | Unfavorable | 46 | 34 |

### **Table S10** *Study 3A Means and Standard Deviations for All Measures across All Cells*

|  | Decision Agent | Decision | Mean | Standard Deviation | |
| --- | --- | --- | --- | --- | --- |
| Fairness | HR Manager | Favorable | 5.35 | | 1.39 |
|  |  | Moderate | 4.21 | | 1.89 |
|  |  | Unfavorable | 3.20 | | 1.65 |
|  | AI System | Favorable | 5.27 | | 1.42 |
|  |  | Moderate | 4.32 | | 1.83 |
|  |  | Unfavorable | 4.14 | | 1.63 |
| Decision Acceptance | HR Manager | Favorable | 6.43 | | .96 |
|  |  | Moderate | 3.94 | | 2.05 |
|  |  | Unfavorable | 2.85 | | 2.08 |
|  | AI System | Favorable | 5.74 | | 1.65 |
|  |  | Moderate | 3.74 | | 2.17 |
|  |  | Unfavorable | 3.09 | | 2.10 |
| Future Engagement | HR Manager | Favorable | 5.78 | | 1.09 |
|  |  | Moderate | 3.24 | | 1.82 |
|  |  | Unfavorable | 2.59 | | 1.55 |
|  | AI System | Favorable | 4.98 | | 1.62 |
|  |  | Moderate | 3.08 | | 1.58 |
|  |  | Unfavorable | 3.01 | | 1.83 |

### **Table S11** *Study 3A Correlations between Variables*

| **Variable** | **1** | **2** |
| --- | --- | --- |
| 1. Fairness |  |  |
| 2. Decision Acceptance | .72** |  |
| 3. Future Engagement | .52** | .56** |

^†^ *p* < .10; ** p < .05; ** p < .01*

## **Study 3B**

### **Table S12** *Study 3B Sample Distribution across Conditions*

| Number of Participants | Conditions | AI System | HR Manager |
| --- | --- | --- | --- |
| Initial *n* | Favorable | 114 | 115 |
|  | Moderate | 128 | 108 |
|  | Unfavorable | 111 | 123 |
| Failed to provide accurate summary of the scenario, failed the manipulation check, or both | Favorable | 18 | 4 |
|  | Moderate | 13 | 2 |
|  | Unfavorable | 10 | 14 |
| Final *n* | Favorable | 96 | 111 |
|  | Moderate | 115 | 106 |
|  | Unfavorable | 101 | 109 |

### **Table S13** *Study 3B Means and Standard Deviations for All Measures across All Cells*

|  | Decision Agent | Decision | Mean | Standard Deviation | |
| --- | --- | --- | --- | --- | --- |
| Fairness | HR Manager | Favorable | 5.00 | | 1.22 |
|  |  | Moderate | 4.63 | | 1.27 |
|  |  | Unfavorable | 3.65 | | 1.46 |
|  | AI System | Favorable | 5.17 | | 1.06 |
|  |  | Moderate | 4.41 | | 1.51 |
|  |  | Unfavorable | 4.16 | | 1.56 |
| Sociability | HR Manager | Favorable | 5.43 | | 1.06 |
|  |  | Moderate | 3.44 | | 1.28 |
|  |  | Unfavorable | 2.45 | | 1.15 |
|  | AI System | Favorable | 4.26 | | 1.45 |
|  |  | Moderate | 2.52 | | 1.37 |
|  |  | Unfavorable | 2.17 | | 1.13 |
| Morality | HR Manager | Favorable | 5.47 | | 1.12 |
|  |  | Moderate | 4.60 | | 1.36 |
|  |  | Unfavorable | 3.61 | | 1.61 |
|  | AI System | Favorable | 4.99 | | 1.26 |
|  |  | Moderate | 4.02 | | 1.59 |
|  |  | Unfavorable | 3.71 | | 1.61 |
| Competence | HR Manager | Favorable | 5.75 | | 1.07 |
|  |  | Moderate | 4.93 | | 1.38 |
|  |  | Unfavorable | 3.99 | | 1.77 |
|  | AI System | Favorable | 5.45 | | 1.32 |
|  |  | Moderate | 4.51 | | 1.63 |
|  |  | Unfavorable | 4.07 | | 1.65 |
| Decision Acceptance | HR Manager | Favorable | 5.59 | | 1.54 |
|  |  | Moderate | 4.42 | | 1.94 |
|  |  | Unfavorable | 2.88 | | 1.86 |
|  | AI System | Favorable | 5.33 | | 1.62 |
|  |  | Moderate | 3.62 | | 1.90 |
|  |  | Unfavorable | 3.06 | | 1.86 |
| Future Engagement | HR Manager | Favorable | 5.51 | | 1.32 |
|  |  | Moderate | 3.81 | | 1.54 |
|  |  | Unfavorable | 3.38 | | 1.75 |
|  | AI System | Favorable | 5.05 | | 1.21 |
|  |  | Moderate | 3.81 | | 1.61 |
|  |  | Unfavorable | 3.30 | | 1.73 |
| Familiarity with AI Decision Making | HR Manager | Favorable | 4.63 | | 1.40 |
|  |  | Moderate | 4.69 | | 1.55 |
|  |  | Unfavorable | 4.29 | | 1.72 |
|  | AI System | Favorable | 4.76 | | 1.47 |
|  |  | Moderate | 4.56 | | 1.73 |
|  |  | Unfavorable | 4.00 | | 1.76 |

### **Table S14** *Study 3B Correlations between Variables*

| **Variable** | **1** | **2** | **3** | **4** | **5** | **6** |
| --- | --- | --- | --- | --- | --- | --- |
| 1. Fairness |  |  |  |  |  |  |
| 2. Sociability | .52** |  |  |  |  |  |
| 3. Morality | .70** | .73** |  |  |  |  |
| 4. Competence | .70** | .64** | .86** |  |  |  |
| 5. Decision Acceptance | .66** | .67** | .73** | .72** |  |  |
| 6. Future Engagement | .40** | .55** | .55** | .55** | .56** |  |
| 7. Familiarity with AI Decision Making | .12** | .14** | .12** | .13** | .13** | .12** |

^†^ *p* < .10; ** p < .05; ** p < .01*

### **Table S15** *Study 3B Moderation Analyses of Decision Agent by Decision on Perceptions of Sociability, Morality, and Competence toward HR Manager vs. AI System*

| Dependent Variable | B | SE | p | 95% CI |
| --- | --- | --- | --- | --- |
| Sociability |  |  |  |  |
| Decision Agent (0 = HR Manager, 1 = AI System) | -.79 | .10 | .0000 | [-.99, -.60] |
| Decision |  |  |  |  |
| W1 (-1 = Favorable, 1 = Moderate, 0 = Unfavorable) | -.33 | .10 | .0007 | [-.53, -.14] |
| W2 (-1 = Favorable, 0 = Moderate, 1 = Unfavorable) | -1.32 | .10 | .0000 | [-1.51, -1.13] |
| Decision Agent $\times$ Decision |  |  |  |  |
| Decision Agent $\times$ W1 | -.13 | .14 | .3633 | [-.40, .15] |
| Decision Agent $\times$ W2 | .51 | .14 | .0003 | [.23, .78] |
| Favorable Decision | -1.17 | .17 | .0000 | [-1.51, -.83] |
| Moderate Decision | -.92 | .17 | .0000 | [-1.25, -.59] |
| Unfavorable Decision | -.29 | .17 | .0945 | [-.62, .05] |
| Morality |  |  |  |  |
| Decision Agent | -.32 | .11 | .0051 | [-.54, -.10] |
| Decision |  |  |  |  |
| W1 | -.04 | .11 | .7356 | [-.18, .26] |
| W2 | -.95 | .11 | .0000 | [-1.17, -.73] |
| Decision Agent $\times$ Decision |  |  |  |  |
| Decision Agent $\times$ W1 | -.25 | .16 | .1131 | [-.57, .06] |
| Decision Agent $\times$ W2 | .42 | .16 | .0095 | [.10, .74] |
| Favorable Decision | -.49 | .20 | .0152 | [-.88, -.09] |
| Moderate Decision | -.57 | .19 | .0032 | [-.95, -.19] |
| Unfavorable Decision | .10 | .20 | .6125 | [-.29, .49] |
| Competence |  |  |  |  |
| Decision Agent | -.21 | .12 | .0712 | [-.45, .02] |
| Decision |  |  |  |  |
| W1 | .04 | .12 | .7139 | [-.19, .27] |
| W2 | -.90 | .12 | .0000 | [-1.13, -.67] |
| Decision Agent $\times$ Decision |  |  |  |  |
| Decision Agent $\times$ W1 | -.21 | .17 | .2055 | [-.54, .12] |
| Decision Agent $\times$ W2 | .29 | .17 | .0854 | [-.04, .62] |

### **Table S16** *Study 3B Moderated Mediation Analyses of Decision Agent by Decision on Decision Acceptance through Fairness as a Mediator, Controlling for Familiarity with AI Decision Making*

|  | Mediator | | |
| --- | --- | --- | --- |
|  | Fairness | | |
|  | B | SE | 95% CI |
| Decision Agent (0 = HR Manager, 1 = AI System) | .16 | .11 | [-.05, .37] |
| Decision |  |  |  |
| W1 (-1 = Favorable, 1 = Moderate, 0 = Unfavorable) | .19^†^ | .11 | [-.02, .40] |
| W2 (-1 = Favorable, 0 = Moderate, 1 = Unfavorable) | -.76*** | .11 | [-.97, -.55] |
| Decision Agent $\times$ Decision |  |  |  |
| Decision Agent $\times$ W1 | -.37* | .15 | [-.67, -.07] |
| Decision Agent $\times$ W2 | .37* | .15 | [.07, .67] |
| Familiarity with AI Decision Making | .07* | .03 | [.007, .14] |
|  | Dependent Variable | | |
|  | Direct Effects on Decision Acceptance | | |
|  | B | SE | 95% CI |
| Decision Agent | -.42*** | .11 | [-.64, -.19] |
| Decision |  |  |  |
| W1 | -.05 | .11 | [-.27, .17] |
| W2 | -.79*** | .12 | [-1.01, -.56] |
| Decision Agent $\times$ Decision |  |  |  |
| Decision Agent $\times$ W1 | -.20 | .16 | [-.52, .11] |
| Decision Agent $\times$ W2 | .19 | .16 | [-.13, .51] |
| Familiarity with AI Decision Making | .02 | .04 | [-.05, .09] |
| Fairness | .80*** | .11 | [.72, .88] |
|  | Indirect Effects on Decision Acceptance | | |
|  | B | SE | 95% CI |
| Indirect Effect through Fairness |  |  |  |
| W1 | -.30 | .12 | [-.54, -.06] |
| W2 | .30 | .13 | [.04, .56] |
| Favorable Decision | .13 | .13 | [-.12, .38] |
| Moderate Decision | -.17 | .15 | [-.46, .13] |
| Unfavorable Decision | .43 | .17 | [.11, .76] |

*Note.* ^†^p < .10; *p<.05; **p<.01; ***p<.001

### **Table S17** *Study 3B Moderated Mediation Analyses of Decision Agent by Decision on Future Engagement through Fairness as a Mediator, Controlling for Familiarity with AI Decision Making*

|  | Mediator | | |
| --- | --- | --- | --- |
|  | Fairness | | |
|  | B | SE | 95% CI |
| Decision Agent (0 = HR Manager, 1 = AI System) | .16 | .11 | [-.05, .37] |
| Decision |  |  |  |
| W1 (-1 = Favorable, 1 = Moderate, 0 = Unfavorable) | .19^†^ | .11 | [-.02, .40] |
| W2 (-1 = Favorable, 0 = Moderate, 1 = Unfavorable) | -.76*** | .11 | [-.97, -.55] |
| Decision Agent $\times$ Decision |  |  |  |
| Decision Agent $\times$ W1 | -.37* | .15 | [-.67, -.07] |
| Decision Agent $\times$ W2 | .37* | .15 | [.07, .67] |
| Familiarity with AI Decision Making | .07* | .03 | [.007, .14] |
|  | Dependent Variable | | |
|  | Direct Effects on Future Engagement | | |
|  | B | SE | 95% CI |
| Decision Agent | -.23* | .12 | [-.46, -.0003] |
| Decision |  |  |  |
| W1 | -.50*** | .12 | [-.73, -.27] |
| W2 | -.57*** | .12 | [-.81, -.34] |
| Decision Agent $\times$ Decision |  |  |  |
| Decision Agent $\times$ W1 | .31^†^ | .16 | [-.01, .63] |
| Decision Agent $\times$ W2 | -.01 | .17 | [-.34, .32] |
| Familiarity with AI Decision Making | .06 | .04 | [-.02, .13] |
| Fairness | .34*** | .04 | [.26, .43] |
|  | Indirect Effects on Future Engagement | | |
|  | B | SE | 95% CI |
| Indirect Effect through Fairness |  |  |  |
| W1 | -.13 | .06 | [-.25, -.02] |
| W2 | .13 | .06 | [.02, .25] |
| Favorable Decision | .05 | .06 | [-.05, .17] |
| Moderate Decision | -.07 | .07 | [-.21, .05] |
| Unfavorable Decision | .18 | .08 | [.04, .34] |

*Note.* ^†^p < .10; *p<.05; **p<.01; ***p<.001

### **Table S18** *Study 3B Moderated Mediation Analyses of Decision Agent by Decision on Decision Acceptance through Fairness and Sociability as Parallel Mediators*

|  | Mediator Variables | | | | | | |  |
| --- | --- | --- | --- | --- | --- | --- | --- | --- |
|  | Fairness | | | Sociability | | | | |
|  | B | SE | | | B | | SE | |
| Decision Agent (0 = HR Manager, 1 = AI System) | .16 | .11 | | | -.79*** | | .10 | |
| Decision |  |  | | |  | |  | |
| W1 (-1 = Favorable, 1 = Moderate, 0 = Unfavorable) | .20^†^ | .11 | | | -.33*** | | .10 | |
| W2 (-1 = Favorable, 0 = Moderate, 1 = Unfavorable) | -.78*** | .11 | | | -1.32*** | | .10 | |
| Decision Agent $\times$ Decision |  |  | | |  | |  | |
| Decision Agent $\times$ W1 | -.37* | .15 | | | -.13 | | .14 | |
| Decision Agent $\times$ W2 | .36* | .15 | | | .51*** | | .14 | |
|  | Dependent Variable | | | | | | |  |
|  | Direct Effects on Decision Acceptance | | | | | | |  |
|  | B | | SE | | | 95% CI | |  |
| Decision Agent | -.03 | | .11 | | | [-.25, .19] | |  |
| Decision |  | |  | | |  | |  |
| W1 | .15 | | .11 | | | [-.07, .36] | |  |
| W2 | -.34** | | .12 | | | [-.58, -.11] | |  |
| Decision Agent $\times$ Decision |  | |  | | |  | |  |
| Decision Agent $\times$ W1 | -.22 | | .15 | | | [-.51, .07] | |  |
| Decision Agent $\times$ W2 | .03 | | .15 | | | [-.27, .32] | |  |
| Fairness | .61*** | | .04 | | | [.52, .70] | |  |
| Sociability | .45*** | | .05 | | | [.36, .55] | |  |
|  | Indirect Effects on Decision Acceptance | | | | | | |  |
|  | B | | SE | | | 95% CI | |  |
| Indirect Effect through Fairness |  | |  | | |  | |  |
| W1 | -.23 | | .10 | | | [-.42, -.05] | |  |
| W2 | .22 | | .10 | | | [.02, .42] | |  |
| Favorable Decision | .10 | | .10 | | | [-.08, .30] | |  |
| Moderate Decision | -.13 | | .11 | | | [-.36, .09] | |  |
| Unfavorable Decision | .31 | | .13 | | | [.06, .58] | |  |
| Indirect Effect through Sociability |  | |  | | |  | |  |
| W1 | -.06 | | .07 | | | [-.19, .07] | |  |
| W2 | .23 | | .07 | | | [.10, .37] | |  |
| Favorable Decision | -.53 | | .11 | | | [-.75, -.33] | |  |
| Moderate Decision | -.42 | | .10 | | | [-.62, -.23] | |  |
| Unfavorable Decision | -.13 | | .07 | | | [-.28, .01] | |  |

*Note.* ^†^p < .10; *p<.05; **p<.01; ***p<.001

### **Table S19** *Study 3B Moderated Mediation Analyses of Decision Agent by Decision on Decision Acceptance through Fairness and Morality as Parallel Mediators*

|  | Mediator Variables | | | | | | |  |
| --- | --- | --- | --- | --- | --- | --- | --- | --- |
|  | Fairness | | | Morality | | | | |
|  | B | SE | | | B | | SE | |
| Decision Agent (0 = HR Manager, 1 = AI System) | .16 | .11 | | | -.32** | | .11 | |
| Decision |  |  | | |  | |  | |
| W1 (-1 = Favorable, 1 = Moderate, 0 = Unfavorable) | .20^†^ | .11 | | | .04 | | .11 | |
| W2 (-1 = Favorable, 0 = Moderate, 1 = Unfavorable) | -.78*** | .11 | | | -.95*** | | .11 | |
| Decision Agent $\times$ Decision |  |  | | |  | |  | |
| Decision Agent $\times$ W1 | -.37* | .15 | | | -.25 | | .16 | |
| Decision Agent $\times$ W2 | .36* | .15 | | | .42** | | .16 | |
|  | Dependent Variable | | | | | | |  |
|  | Direct Effects on Decision Acceptance | | | | | | |  |
|  | B | | SE | | | 95% CI | |  |
| Decision Agent | -.17 | | .10 | | | [-.37, .03] | |  |
| Decision |  | |  | | |  | |  |
| W1 | .02 | | .10 | | | [-.19, .21] | |  |
| W2 | -.55*** | | .11 | | | [-.76, -.34] | |  |
| Decision Agent $\times$ Decision |  | |  | | |  | |  |
| Decision Agent $\times$ W1 | -.21 | | .14 | | | [-.49, .07] | |  |
| Decision Agent $\times$ W2 | .08 | | .14 | | | [-.20, .37] | |  |
| Fairness | .40*** | | .05 | | | [.30, .50] | |  |
| Morality | .58*** | | .05 | | | [.49, .67] | |  |
|  | Indirect Effects on Decision Acceptance | | | | | | |  |
|  | B | | SE | | | 95% CI | |  |
| Indirect Effect through Fairness |  | |  | | |  | |  |
| W1 | -.15 | | .07 | | | [-.28, -.03] | |  |
| W2 | .14 | | .07 | | | [.02, .29] | |  |
| Favorable Decision | .07 | | .06 | | | [-.06, .20] | |  |
| Moderate Decision | -.09 | | .08 | | | [-.23, .06] | |  |
| Unfavorable Decision | .20 | | .09 | | | [.04, .39] | |  |
| Indirect Effect through Morality |  | |  | | |  | |  |
| W1 | -.15 | | .09 | | | [-.34, .04] | |  |
| W2 | .24 | | .10 | | | [.05, .46] | |  |
| Favorable Decision | -.28 | | .10 | | | [-.49, -.10] | |  |
| Moderate Decision | -.33 | | .12 | | | [-.58, -.11] | |  |
| Unfavorable Decision | .06 | | .13 | | | [-.20, .31] | |  |

*Note.* ^†^p < .10; *p<.05; **p<.01; ***p<.001

### **Table S20** *Study 3B Moderated Mediation Analyses of Decision Agent by Decision on Decision Acceptance through Fairness and Competence as Parallel Mediators*

|  | Mediator Variables | | | | | | |  |
| --- | --- | --- | --- | --- | --- | --- | --- | --- |
|  | Fairness | | | Competence | | | | |
|  | B | SE | | | B | | SE | |
| Decision Agent (0 = HR Manager, 1 = AI System) | .16 | .11 | | | -.21^†^ | | .12 | |
| Decision |  |  | | |  | |  | |
| W1 (-1 = Favorable, 1 = Moderate, 0 = Unfavorable) | .20^†^ | .11 | | | .04 | | .12 | |
| W2 (-1 = Favorable, 0 = Moderate, 1 = Unfavorable) | -.78*** | .11 | | | -.90*** | | .12 | |
| Decision Agent $\times$ Decision |  |  | | |  | |  | |
| Decision Agent $\times$ W1 | -.37* | .15 | | | -.21 | | .17 | |
| Decision Agent $\times$ W2 | .36* | .15 | | | .29^†^ | | .17 | |
|  | Dependent Variable | | | | | | |  |
|  | Direct Effects on Decision Acceptance | | | | | | |  |
|  | B | | SE | | | 95% CI | |  |
| Decision Agent | -.24* | | .10 | | | [-.44, -.04] | |  |
| Decision |  | |  | | |  | |  |
| W1 | .01 | | .10 | | | [-.19, .21] | |  |
| W2 | -.61*** | | .11 | | | [-.81, -.40] | |  |
| Decision Agent $\times$ Decision |  | |  | | |  | |  |
| Decision Agent $\times$ W1 | -.24 | | .14 | | | [-.52, .04] | |  |
| Decision Agent $\times$ W2 | .17 | | .15 | | | [-.12, .45] | |  |
| Fairness | .41*** | | .05 | | | [.31, .51] | |  |
| Competence | .55*** | | .05 | | | [.46, .64] | |  |
|  | Indirect Effects on Decision Acceptance | | | | | | |  |
|  | B | | SE | | | 95% CI | |  |
| Indirect Effect through Fairness |  | |  | | |  | |  |
| W1 | -.15 | | .07 | | | [-.29, -.03] | |  |
| W2 | .15 | | .07 | | | [.02, .29] | |  |
| Favorable Decision | .07 | | .07 | | | [-.06, .20] | |  |
| Moderate Decision | -.09 | | .08 | | | [-.25, .06] | |  |
| Unfavorable Decision | .21 | | .09 | | | [.04, .40] | |  |
| Indirect Effect through Competence |  | |  | | |  | |  |
| W1 | -.11 | | .09 | | | [-.29, .07] | |  |
| W2 | .16 | | .10 | | | [-.03, .35] | |  |
| Favorable Decision | -.16 | | .09 | | | [-.35, .01] | |  |
| Moderate Decision | -.23 | | .11 | | | [-.45, -.01] | |  |
| Unfavorable Decision | .04 | | .13 | | | [-.21, .29] | |  |

*Note.* ^†^p < .10; *p<.05; **p<.01; ***p<.001

Results from all three moderated mediation analyses on decision acceptance with simultaneous mediators (Tables S11, S12, and S13) show that there is a significant indirect effect of Decision Agent on decision acceptance through perceived fairness when the decision is unfavorable; when the decision is unfavorable, perceived fairness of AI is higher, leading to higher decision acceptance. Thus, the hypothesized effects are supported.

Results from the moderated mediation analyses with sociability and fairness as simultaneous mediators (Table S11) show that there is a significant indirect effect of Decision Agent on decision acceptance through perceived sociability when the decision is favorable or moderate; when the decision is favorable or moderate, perceived sociability of AI is lower, leading to lower decision acceptance. The moderated mediation analyses with morality and fairness as simultaneous mediators (Table S12) also show that there is a significant indirect effect of Decision Agent on decision acceptance through perceived morality under favorable and moderate decision conditions; in the face of favorable or moderate decisions, individuals perceive AI as less moral, and are less likely to accept the decisions from AI. There was no significant indirect effect through competence (Table S13). As the perceived sociability, morality, and competence of decision agent are not in the scope of the current paper, the results are not further discussed. Future studies may examine the roles of perceived sociability and morality in addition to fairness in the relationship between decision agent and decision acceptance, especially when the decisions are favorable.

### **Table S21** *Study 3B Moderated Mediation Analyses of Decision Agent by Decision on Future Engagement through Fairness and Sociability as Parallel Mediators*

|  | Mediator Variables | | | | | | |  |
| --- | --- | --- | --- | --- | --- | --- | --- | --- |
|  | Fairness | | | Sociability | | | | |
|  | B | SE | | | B | | SE | |
| Decision Agent (0 = HR Manager, 1 = AI System) | .16 | .11 | | | -.79*** | | .10 | |
| Decision |  |  | | |  | |  | |
| W1 (-1 = Favorable, 1 = Moderate, 0 = Unfavorable) | .20^†^ | .11 | | | -.33*** | | .10 | |
| W2 (-1 = Favorable, 0 = Moderate, 1 = Unfavorable) | -.78*** | .11 | | | -1.32*** | | .10 | |
| Decision Agent $\times$ Decision |  |  | | |  | |  | |
| Decision Agent $\times$ W1 | -.37* | .15 | | | -.13 | | .14 | |
| Decision Agent $\times$ W2 | .36* | .15 | | | .51*** | | .14 | |
|  | Dependent Variable | | | | | | |  |
|  | Direct Effects on Future Engagement | | | | | | |  |
|  | B | | SE | | | 95% CI | |  |
| Decision Agent | .08 | | .12 | | | [-.16, .31] | |  |
| Decision |  | |  | | |  | |  |
| W1 | -.34** | | .11 | | | [-.56, -.12] | |  |
| W2 | -.23^†^ | | .13 | | | [-.47, .02] | |  |
| Decision Agent $\times$ Decision |  | |  | | |  | |  |
| Decision Agent $\times$ W1 | .30^†^ | | .16 | | | [-.01, .61] | |  |
| Decision Agent $\times$ W2 | -.15 | | .16 | | | [-.47, .17] | |  |
| Fairness | .19*** | | .05 | | | [.10, .28] | |  |
| Sociability | .36*** | | .05 | | | [.26, .46] | |  |
|  | Indirect Effects on Future Engagement | | | | | | |  |
|  | B | | SE | | | 95% CI | |  |
| Indirect Effect through Fairness |  | |  | | |  | |  |
| W1 | -.07 | | .04 | | | [-.15, -.01] | |  |
| W2 | .07 | | .04 | | | [.007, .15] | |  |
| Favorable Decision | .03 | | .03 | | | [-.03, .11] | |  |
| Moderate Decision | -.04 | | .04 | | | [-.13, .03] | |  |
| Unfavorable Decision | .10 | | .05 | | | [.02, .21] | |  |
| Indirect Effect through Sociability |  | |  | | |  | |  |
| W1 | -.05 | | .05 | | | [-.16, .05] | |  |
| W2 | .18 | | .05 | | | [.08, .29] | |  |
| Favorable Decision | -.42 | | .09 | | | [-.61, -.26] | |  |
| Moderate Decision | -.33 | | .08 | | | [-.52, -.18] | |  |
| Unfavorable Decision | -.10 | | .06 | | | [-.23, .004] | |  |

*Note.* ^†^p < .10; *p<.05; **p<.01; ***p<.001

### **Table S22** *Study 3B Moderated Mediation Analyses of Decision Agent by Decision on Future Engagement through Fairness and Morality as Parallel Mediators*

|  | Mediator Variables | | | | | | |  |
| --- | --- | --- | --- | --- | --- | --- | --- | --- |
|  | Fairness | | | Morality | | | | |
|  | B | SE | | | B | | SE | |
| Decision Agent (0 = HR Manager, 1 = AI System) | .16 | .11 | | | -.32** | | .11 | |
| Decision |  |  | | |  | |  | |
| W1 (-1 = Favorable, 1 = Moderate, 0 = Unfavorable) | .20^†^ | .11 | | | .04 | | .11 | |
| W2 (-1 = Favorable, 0 = Moderate, 1 = Unfavorable) | -.78*** | .11 | | | -.95*** | | .11 | |
| Decision Agent $\times$ Decision |  |  | | |  | |  | |
| Decision Agent $\times$ W1 | -.37* | .15 | | | -.25 | | .16 | |
| Decision Agent $\times$ W2 | .36* | .15 | | | .42** | | .16 | |
|  | Dependent Variable | | | | | | |  |
|  | Direct Effects on Future Engagement | | | | | | |  |
|  | B | | SE | | | 95% CI | |  |
| Decision Agent | -.03 | | .11 | | | [-.25, .19] | |  |
| Decision |  | |  | | |  | |  |
| W1 | -.44*** | | .11 | | | [-.66, -.23] | |  |
| W2 | -.39*** | | .11 | | | [-.62, -.17] | |  |
| Decision Agent $\times$ Decision |  | |  | | |  | |  |
| Decision Agent $\times$ W1 | .31* | | .15 | | | [.002, .61] | |  |
| Decision Agent $\times$ W2 | -.10 | | .16 | | | [-.41, .20] | |  |
| Fairness | .02 | | .05 | | | [-.09, .12] | |  |
| Morality | .47*** | | .05 | | | [.37, .57] | |  |
|  | Indirect Effects on Future Engagement | | | | | | |  |
|  | B | | SE | | | 95% CI | |  |
| Indirect Effect through Fairness |  | |  | | |  | |  |
| W1 | -.006 | | .02 | | | [-.06, .04] | |  |
| W2 | .006 | | .02 | | | [-.04, .05] | |  |
| Favorable Decision | .003 | | .01 | | | [-.02, .03] | |  |
| Moderate Decision | -.004 | | .02 | | | [-.04, .03] | |  |
| Unfavorable Decision | .008 | | .03 | | | [-.05, .07] | |  |
| Indirect Effect through Morality |  | |  | | |  | |  |
| W1 | -.12 | | .08 | | | [-.27, .03] | |  |
| W2 | .20 | | .08 | | | [.04, .37] | |  |
| Favorable Decision | -.23 | | .10 | | | [-.41, -.07] | |  |
| Moderate Decision | -.27 | | .10 | | | [-.47, -.08] | |  |
| Unfavorable Decision | .05 | | .11 | | | [-.16, .25] | |  |

*Note.* ^†^p < .10; *p<.05; **p<.01; ***p<.001

### **Table S23** *Study 3B Moderated Mediation Analyses of Decision Agent by Decision on Future Engagement through Fairness and Competence as Parallel Mediators*

|  | Mediator Variables | | | | | | |  |
| --- | --- | --- | --- | --- | --- | --- | --- | --- |
|  | Fairness | | | Competence | | | | |
|  | B | SE | | | B | | SE | |
| Decision Agent (0 = HR Manager, 1 = AI System) | .16 | .11 | | | -.21^†^ | | .12 | |
| Decision |  |  | | |  | |  | |
| W1 (-1 = Favorable, 1 = Moderate, 0 = Unfavorable) | .20^†^ | .11 | | | .04 | | .12 | |
| W2 (-1 = Favorable, 0 = Moderate, 1 = Unfavorable) | -.78*** | .11 | | | -.90*** | | .12 | |
| Decision Agent $\times$ Decision |  |  | | |  | |  | |
| Decision Agent $\times$ W1 | -.37* | .15 | | | -.21 | | .17 | |
| Decision Agent $\times$ W2 | .36* | .15 | | | .29^†^ | | .17 | |
|  | Dependent Variable | | | | | | |  |
|  | Direct Effects on Future Engagement | | | | | | |  |
|  | B | | SE | | | 95% CI | |  |
| Decision Agent | -.08 | | .11 | | | [-.30, .13] | |  |
| Decision |  | |  | | |  | |  |
| W1 | -.44*** | | .11 | | | [-.66, -.23] | |  |
| W2 | -.43*** | | .11 | | | [-.65,-.20 ] | |  |
| Decision Agent $\times$ Decision |  | |  | | |  | |  |
| Decision Agent $\times$ W1 | .28^†^ | | .15 | | | [-.02, .58] | |  |
| Decision Agent $\times$ W2 | -.04 | | .16 | | | [-.34, .27] | |  |
| Fairness | .007 | | .05 | | | [-.10, .11] | |  |
| Competence | .47*** | | .05 | | | [.37, .57] | |  |
|  | Indirect Effects on Future Engagement | | | | | | |  |
|  | B | | SE | | | 95% CI | |  |
| Indirect Effect through Fairness |  | |  | | |  | |  |
| W1 | -.003 | | .02 | | | [-.06, .04] | |  |
| W2 | .003 | | .02 | | | [-.04, .05] | |  |
| Favorable Decision | .001 | | .02 | | | [-.03, .04] | |  |
| Moderate Decision | -.002 | | .02 | | | [-.04, .03] | |  |
| Unfavorable Decision | .004 | | .04 | | | [-.06, .07] | |  |
| Indirect Effect through Competence |  | |  | | |  | |  |
| W1 | -.10 | | .08 | | | [-.25, .02] | |  |
| W2 | .14 | | .08 | | | [-.03, .30] | |  |
| Favorable Decision | -.14 | | .08 | | | [-.30, .02] | |  |
| Moderate Decision | -.20 | | .10 | | | [-.40, -.02] | |  |
| Unfavorable Decision | .04 | | .11 | | | [-.18, .25] | |  |
| *Note.* ^†^p < .10; *p<.05; **p<.01; ***p<.001 |  | |  | | |  | |  |

Results from the moderated mediation analyses on future engagement with fairness and sociability simultaneous mediators (Tables S14) show that there is a significant indirect effect of Decision Agent on future engagement through perceived fairness when the decision is unfavorable; when the decision is unfavorable, perceived fairness of AI is higher, leading to higher future engagement. Therefore, the hypothesized effects are supported. Results from the same analyses show that there is a significant indirect effect of Decision Agent on future engagement through perceived sociability when the decision is favorable or moderate; when the decision is favorable or moderate, perceived sociability of AI is lower, leading to lower future engagement.

Results from the moderated mediation analyses on future engagement with fairness and morality simultaneous mediators (Tables S15) show that there is a pattern for a positive indirect effect of Decision Agent on future engagement through perceived fairness under the Unfavorable Decision Condition, which is consistent with the hypothesized effects. There is a significant indirect effect of Decision Agent on future engagement through perceived morality under favorable and moderate decision conditions; in the face of favorable or moderate decisions, individuals perceive AI as less moral, and less likely to exhibit future engagement.

Results from the moderated mediation analyses on future engagement with fairness and morality simultaneous mediators (Tables S16) also show that there is a consistent pattern for a positive indirect effect of Decision Agent on future engagement through perceived fairness under the Unfavorable Decision Condition. There was no significant indirect effect through competence.

We do not further discuss the results as the perceived sociability, morality, and competence of decision agent are not in the scope of the current paper. Future studies may examine the roles of perceived sociability and morality, competence in addition to fairness in the relationship between decision agent and future engagement, especially in the context of favorable decisions.

### **Table S24** *Studies 3A and 3B Combined Moderated Mediation Analyses of Decision Agent by Decision on Decision Acceptance through Fairness as a Mediator*

|  | Mediator | | |
| --- | --- | --- | --- |
|  | Fairness | | |
|  | B | SE | 95% CI |
| Decision Agent (0 = HR Manager, 1 = AI System) | .21* | .10 | [.02, .40] |
| Decision |  |  |  |
| W1 (-1 = Favorable, 1 = Moderate, 0 = Unfavorable) | .13 | .10 | [-.06, .31] |
| W2 (-1 = Favorable, 0 = Moderate, 1 = Unfavorable) | -.84*** | .10 | [-1.03, -.64] |
| Decision Agent $\times$ Decision |  |  |  |
| Decision Agent $\times$ W1 | -.32* | .14 | [-.59, -.05] |
| Decision Agent $\times$ W2 | .41** | .14 | [.14, .68] |
| Study (0 = 3A, 1 = 3B) | .09 | .11 | [-.12, .31] |
|  | Dependent Variable | | |
|  | Direct Effects on Decision Acceptance | | |
|  | B | SE | 95% CI |
| Decision Agent | -.42*** | .10 | [-.61, -.24] |
| Decision |  |  |  |
| W1 | -.16^†^ | .09 | [-.34, .03] |
| W2 | -.77*** | .07 | [-.85, -.57] |
| Decision Agent $\times$ Decision |  |  |  |
| Decision Agent $\times$ W1 | -.09 | .13 | [-.35, .17] |
| Decision Agent $\times$ W2 | .11 | .14 | [-.15, .38] |
| Fairness | .80*** | .03 | [.73, .86] |
| Study (0 = 3A, 1 = 3B) | -.20^†^ | .11 | [-.41, .005] |
|  | Indirect Effects on Decision Acceptance | | |
|  | B | SE | 95% CI |
| Indirect Effect through Fairness |  |  |  |
| W1 | -.26 | .11 | [-.48, -.03] |
| W2 | .33 | .11 | [.11, .55] |
| Favorable Decision | .09 | .12 | [-.14, .33] |
| Moderate Decision | -.09 | .14 | [-.37, .19] |
| Unfavorable Decision | .50 | .15 | [.21, .79] |

*Note.* ^†^p < .10; *p<.05; **p<.01; ***p<.001

### **Table S25** *Studies 3A and 3B Moderated Mediation Analyses of Decision Agent by Decision on Future Engagement through Fairness as a Mediator*

|  | Mediator | | |
| --- | --- | --- | --- |
|  | Fairness | | |
|  | B | SE | 95% CI |
| Decision Agent (0 = HR Manager, 1 = AI System) | .21* | .10 | [.02, .40] |
| Decision |  |  |  |
| W1 (-1 = Favorable, 1 = Moderate, 0 = Unfavorable) | .13 | .10 | [-.06, .31] |
| W2 (-1 = Favorable, 0 = Moderate, 1 = Unfavorable) | -.84*** | .10 | [-1.03, -.64] |
| Decision Agent $\times$ Decision |  |  |  |
| Decision Agent $\times$ W1 | -.32* | .14 | [-.59, -.05] |
| Decision Agent $\times$ W2 | .41** | .14 | [.14, .68] |
| Study (0 = 3A, 1 = 3B) | .09 | .11 | [-.12, .31] |
|  | Dependent Variable | | |
|  | Direct Effects on Future Engagement | | |
|  | B | SE | 95% CI |
| Decision Agent | -.25* | .10 | [-.45, -.06] |
| Decision |  |  |  |
| W1 | -.53*** | .10 | [-.72, -.34] |
| W2 | -.64*** | .10 | [-.84, -.43] |
| Decision Agent $\times$ Decision |  |  |  |
| Decision Agent $\times$ W1 | .26 | .14 | [-.009, .54] |
| Decision Agent $\times$ W2 | .06 | .14 | [-.22, .34] |
| Fairness | .38*** | .03 | [.31, .44] |
| Study (0 = 3A, 1 = 3B) | .33** | .11 | [.11, .55] |
|  | Indirect Effects on Future Engagement | | |
|  | B | SE | 95% CI |
| Indirect Effect through Fairness |  |  |  |
| W1 | -.12 | .06 | [-.23, -.02] |
| W2 | .16 | .06 | [.05, .27] |
| Favorable Decision | .04 | .06 | [-.06, .16] |
| Moderate Decision | -.04 | .07 | [-.18, .09] |
| Unfavorable Decision | .23 | .07 | [.10, .38] |

*Note.* ^†^p < .10; *p<.05; **p<.01; ***p<.001

## **Study 4**

### **Table S26** *Study 4 Sample Distribution across Conditions*

| Number of Participants | AI Assistant | Research Assistant |
| --- | --- | --- |
| Initial *n* | 107 | 135 |
| Failed to follow instructions | 6 | 18 |
| Final *n* | 101 | 117 |

### **Table S27** *Study 4 Means and Standard Deviations for All Measures across All Cells*

|  | Decision Agent | Mean | Standard Deviation | |
| --- | --- | --- | --- | --- |
| Objectivity | HR Manager | 4.26 | | 1.58 |
|  | AI System | 4.57 | | 1.63 |
| Unemotionality | HR Manager | 4.63 | | 1.72 |
|  | AI System | 5.31 | | 1.59 |
| Fairness | HR Manager | 4.13 | | 1.37 |
|  | AI System | 4.48 | | 1.22 |
| Initial Performance | HR Manager | 223.98 | | 46.58 |
|  | AI System | 225.67 | | 43.24 |
| Final Performance | HR Manager | 248.55 | | 39.76 |
|  | AI System | 250.87 | | 40.93 |

### **Table S28** *Study 4 Correlations between Variables*

| **Variable** | **1** | **2** | **3** | **4** |
| --- | --- | --- | --- | --- |
| 1. Objectivity |  |  |  |  |
| 2. Unemotionality | .65** |  |  |  |
| 3. Fairness | .81** | .61** |  |  |
| 4. Initial Performance | .04 | .07** | -.03 |  |
| 5. Final Performance | .13^†^ | .14* | .11 | .62** |

^†^ *p* < .10; ** p < .05; ** p < .01*

## **Study 5**

### **Table S29** *Study 5 Sample Distribution across Conditions*

| Number of Participants | AI System | AI Bias | HR Manager |
| --- | --- | --- | --- |
| Initial *n* | 404 | 397 | 407 |
| Failed to provide accurate summary of the scenario, failed the manipulation check, or both | 43 | 34 | 39 |
| Final *n* | 361 | 363 | 368 |

### **Table S30** *Study 5 Means and Standard Deviations for All Measures across All Cells*

|  | Condition | Mean | Standard Deviation | |  |
| --- | --- | --- | --- | --- | --- |
| Fairness | HR Manager | 3.11 | | 1.38 |  |
|  | AI Bias | 3.27 | | 1.45 |  |
|  | AI System | 3.40 | | 1.38 |  |
| Sociability | HR Manager | 2.68 | | 1.19 |  |
|  | AI Bias | 2.33 | | 1.12 |  |
|  | AI System | 2.22 | | 1.10 |  |
| Morality | HR Manager | 2.98 | | 1.46 |  |
|  | AI Bias | 2.94 | | 1.48 |  |
|  | AI System | 2.83 | | 1.41 |  |
| Competence | HR Manager | 3.21 | | 1.50 |  |
|  | AI Bias | 3.31 | | 1.60 |  |
|  | AI System | 2.92 | | 1.53 |  |
| Decision Acceptance | HR Manager | 3.67 | | 1.66 |  |
|  | AI Bias | 3.71 | | 1.60 |  |
|  | AI System | 3.53 | | 1.57 |  |
| Future Engagement | HR Manager | 2.75 | | 1.43 |  |
|  | AI Bias | 2.75 | | 1.37 |  |
|  | AI System | 2.74 | | 1.36 |  |
| Familiarity with AI Decision Making | | HR Manager | 4.57 | | 1.40 |
|  |  | AI Bias | 4.39 | | 1.52 |
|  |  | AI System | 4.33 | | 1.53 |

### **Table S31** *Study 5 Correlations between Variables*

| **Variable** | **1** | **2** | **3** | **4** | **5** | **6** |
| --- | --- | --- | --- | --- | --- | --- |
| 1. Fairness |  |  |  |  |  |  |
| 2. Sociability | .54** |  |  |  |  |  |
| 3. Morality | .74** | .71** |  |  |  |  |
| 4. Competence | .70** | .67** | .84** |  |  |  |
| 5. Decision Acceptance | .62** | .51** | .67** | .66** |  |  |
| 6. Future Engagement | .41** | .52** | .52** | .51** | .47** |  |
| 7. Familiarity with AI Decision Making | .007 | .07* | .08* | .07* | .02 | .10** |

^†^ *p* < .10; ** p < .05; ** p < .01*

### **Table S32** *Study 5 Mediation Analyses of Decision Agent on Decision Acceptance and Future Engagement through Fairness as a Mediator, Controlling for Familiarity with AI Decision Making*

|  | B | | SE | 95% CI | |
| --- | --- | --- | --- | --- | --- |
|  |  |  |  | LL | UL |
| **Decision Agent 🡪 Fairness 🡪 Decision Acceptance** | | | | | |
| X1 (HR Manager = -1; AI System = 1) | .10 | .04 | | .02 | .18 |
| X2 (HR Manager = -1; AI Bias = 1) | .008 | .04 | | -.08 | .09 |
| **Decision Agent 🡪 Fairness 🡪 Future Engagement** | | | | | |
| X1 (HR Manager = -1; AI System = 1) | .06 | .03 | | .01 | .11 |
| X2 (HR Manager = -1; AI Bias = 1) | .005 | .03 | | -.04 | .05 |

### ***Study 5 Results on Sociability, Morality, and Competence***

To examine the effect of Condition on the perceived sociability, morality, and competence of the decision agent, we ran a General Linear Model with Condition as an independent variable, and sociability, morality, and competence as dependent variables. Results showed that there was a significant effect of Condition on sociability, *F* (2, 1089) = 16.08, *p < .*001, $\eta$*_p_^2^* = .029, and competence, *F* (2, 1089) = 6.20, *p = .*002, $\eta$*_p_^2^* = .011, but not on morality, *F* (2, 1089) = 1.07, *p = .*344, $\eta$*_p_^2^* = .002. Results on the pairwise comparisons (Table S33) showed that the AI system was perceived as significantly less sociable than the HR Manager, and individuals who were reminded of AI biases still perceived the AI system as significantly less sociable than the HR manager. Results further showed that the AI system was perceived significantly less competent than the HR manager, but individuals who learned about AI’s capacity to replicate human biases did not differentiate between the AI system and the HR manager in terms of competence.

We do not further discuss the results as the perceived sociability, morality, and competence of decision agent are not in the scope of the current paper. Future studies may examine the roles of perceived sociability and morality, competence in addition to fairness in the relationship between decision agent and future engagement, especially when individuals have information about AI’s capacity to replicate human biases.

**Table S33** *Study 5 Pairwise Comparisons on Sociability, Morality, and Competence*

| Dependent Variable | (I) Condition | (J) Condition | Mean Difference (I – J) | *SE* | *p* | 95% CI |
| --- | --- | --- | --- | --- | --- | --- |
| Sociability |  |  |  |  |  |  |
|  | HR Manager | AI System | .46 | .08 | .000 | [.29, .62] |
|  | HR Manager | AI Bias | .34 | .08 | .000 | [.18, .51] |
|  | AI System | AI Bias | -.11 | .09 | .176 | [-.28, .05] |
| Morality |  |  |  |  |  |  |
|  | HR Manager | AI System | .15 | .11 | .158 | [-.06, .36] |
|  | HR Manager | AI Bias | .04 | .11 | .705 | [-.17, .25] |
|  | AI System | AI Bias | -.11 | .11 | .303 | [-.32, .10] |
| Competence |  |  |  |  |  |  |
|  | HR Manager | AI System | .29 | .11 | .010 | [.07, .52] |
|  | HR Manager | AI Bias | -.09 | .11 | .416 | [-.32, .13] |
|  | AI System | AI Bias | -.38 | .12 | .000 | [-.61, -.16] |

## **Supplemental Study**

We conducted a supplemental study with the following three objectives. First, we aimed to rule out the potential confounding effect of the manipulation used in our studies. From Studies 1 to 3B, we manipulated the decision agent such that either “the AI system that has been trained with 10 years’ worth of personnel data” or “the HR manager who has 10 years of experience in handling personnel issues” would make a final decision. This manipulation might signal that the AI system and the HR manager have access to different amount of data. Therefore, in this study, we tested the effect of decision agent (AI vs. Human) and decision outcome (Favorable vs. Unfavorable) on fairness and reactions to the decision with a modification in our experimental manipulation which showed that the AI system and HR manager had access to the same amount of data. Second, we aimed to test the alternative mechanisms behind the effect of decision agent and decision on reactions towards the decision. Specifically, we wanted to test perceived control over decisions and typicality of decision making as potential alternative mediators. If individuals feel that they have less control over the unfavorable decisions made by AI, they might be more likely to accept those decisions from AI. Moreover, if they consider unfavorable decisions being made by AI (vs. human) as less typical, they might more willingly accept the decisions from AI as they are less familiar with algorithmic decision making. Lastly, the main study examined the effect of decision agent (AI vs. Human) and decision outcome (Favorable vs. Unfavorable) on such reactions as decision acceptance and future engagement. These outcomes were both relatively positive from the organization perspective. To explore whether the effects would be generalized to reactions that would have negative organizational implications, we investigated the impact of decision agent and decision outcome on organizational deviance in the supplemental study. As perceived control, typicality and organizational deviance are not within the scope of the current paper, the study is not reported in the manuscript but is reported below for readers who might wonder about the alternative mechanisms and generalizability of the effects to different kind of outcomes. The full survey materials and data of the supplemental study in the following OSF page: <https://osf.io/h4euc/?view_only=7ebc7de005d94af29b8bc8d1487d2f35>.

### ***Method***

**Participants.** Eight hundred and one participants were recruited the same way as in Study 2. After excluding 72 participants who failed to accurately summarize the scenario, or failed the manipulation check, or failed both, 729 participants remained in the final sample (63.2% Europeans, 32.4% Africans, 1.4% Asians, 1.4 % North Americans, 1.1% South Americans, and .5% Australians; 364 female; age: *M* = 29.85 years, *SD* = 13.90).

**Design and Procedure.** Participants were randomly assigned to one of the 2 (Decision Agent: HR Manager vs. AI System) $\times$ 2 (Decision: Favorable vs. Unfavorable) between-subjects conditions. This study adopted the same design and procedure as in Study 2 except the following modification in the Decision Agent manipulation in the scenario. Specifically, participants read that, “[their] case was input into an Artificial Intelligence system (vs. passed on to a Human Resources manager) with access to 10 years' worth of personnel data.” After reading the scenario, they provided a summary of the scenario and evaluated perceived control over the decision using a three-item scale (Johnson et al., 2002; α = .88; 1 = Not likely at all; 7 = Extremely likely; e.g., “To what extent do you think you could have control over how the AI system (vs. HR manager) makes the final decision?”) and perceived typicality using a one-item scale (Newman et al., 2020; 0 = Not at all typical; 100 = Completely typical; “In your opinion, how typical is this company's conflict resolution process on a scale of 0 to100?”). They also evaluated the perceived fairness of the decision agent (α = .81), decision acceptance, and future engagement (α = .91). They were also asked to indicate the extent to which they would engage in deviant behaviors (Bennet & Robinson, 2000; α = .91) on a twelve-item measure using a seven-point scale (1 = Not likely at all; 7 = Extremely likely). They were asked to recall the decision agent and decision in the scenario as a manipulation check.

### ***Results***

See Table S30 for the means and standard deviations for all measures across all cells and Table S31 for correlations between all measures.

**Table S34** *Supplemental Study Means and Standard Deviations for All Measures across All Cells*

|  | Decision Agent | Decision | Mean | Standard Deviation | |
| --- | --- | --- | --- | --- | --- |
| Typicality | HR Manager | Favorable | 61.54 | | 22.25 |
|  |  | Unfavorable | 57.98 | | 25.45 |
|  | AI System | Favorable | 39.39 | | 29.77 |
|  |  | Unfavorable | 29.94 | | 26.87 |
| Control | HR Manager | Favorable | 5.26 | | 1.33 |
|  |  | Unfavorable | 3.27 | | 1.30 |
|  | AI System | Favorable | 2.68 | | 1.50 |
|  |  | Unfavorable | 2.68 | | 1.49 |
| Fairness | HR Manager | Favorable | 5.26 | | 1.33 |
|  |  | Unfavorable | 3.27 | | 1.30 |
|  | AI System | Favorable | 5.12 | | 1.38 |
|  |  | Unfavorable | 3.59 | | 1.43 |
| Decision Acceptance | HR Manager | Favorable | 6.53 | | 1.55 |
|  |  | Unfavorable | 3.80 | | 1.69 |
|  | AI System | Favorable | 6.24 | | 1.70 |
|  |  | Unfavorable | 3.61 | | 1.53 |
| Future Engagement | HR Manager | Favorable | 4.26 | | 1.33 |
|  |  | Unfavorable | 3.07 | | 1.50 |
|  | AI System | Favorable | 4.11 | | 1.43 |
|  |  | Unfavorable | 3.25 | | 1.35 |
| Organizational Deviance | HR Manager | Favorable | 1.78 | | .94 |
|  |  | Unfavorable | 2.34 | | 1.14 |
|  | AI System | Favorable | 1.97 | | 1.04 |
|  |  | Unfavorable | 2.11 | | .97 |
| Familiarity with AI Decision Making | HR Manager | Favorable | 4.45 | | 1.71 |
|  |  | Unfavorable | 4.49 | | 1.63 |
|  | AI System | Favorable | 4.33 | | 1.65 |
|  |  | Unfavorable | 3.94 | | 1.59 |

**Table S35** *Supplemental Study Correlations between Variables*

| **Variable** | **1** | **2** | **3** | **4** | **5** | **6** |
| --- | --- | --- | --- | --- | --- | --- |
| 1. Typicality |  |  |  |  |  |  |
| 2. Control | .02 |  |  |  |  |  |
| 3. Fairness | .08* | -.13** |  |  |  |  |
| 4. Decision Acceptance | .15** | -.11** | .70** |  |  |  |
| 5. Future Engagement | .13* | .10** | .30** | .39** |  | . |
| 6. Organizational Deviance | -.02 | .17** | -.17** | -.17** | -.26** |  |
| 7. Familiarity with AI Decision Making | .09* | .09* | .04 | .02 | .14** | -.05 |

^†^ *p* < .10; ** p < .05; ** p < .01*

**Alternative Mechanisms.** We used PROCESS Model 1(Hayes 2018, 5,000 bootstrap samples) to test the impact of Decision Agent and Decision on perceived control and typicality. In the model, decision agent (0 = HR Manager, 1 = AI System) was entered as an independent variable, decision (0 = Favorable, 1 = Unfavorable) was entered as a moderator, and perceived control and typicality were separately entered as a dependent variable.

***Perceived Control.*** Results revealed a significant main effect of Decision Agent, *B* = -.37, *SE* = .15, *p* = .0132, 95% CI [-.66, -.08]; perceived control was significantly lower when the decision agent was AI (vs. human). The main effect of Decision, *B* = -.12, *SE* = .15, *p* = .4315, 95% CI [-.42, .18], and the Decision Agent by Decision interaction on perceived control were not significant, *B* = .13, *SE* = .21, *p* = .5539, 95% CI [-.29, .54].

***Perceived Typicality.*** Results also showed that there was a significant main effect of Decision Agent on perceived typicality, with the typicality was rated significantly lower when the decision agent was AI (vs. human)*, B* = -.22.15, *SE* = 2.73, *p* < .001, 95% CI [-27.52, -16.78]. There was no main effect of Decision, *B* = -3.56, *SE* = 2.80, *p* = .2050, 95% CI [-9.06, 1.95], or the Decision Agent $\times$ Decision interaction on perceived typicality, *B* = -5.89, *SE* = 3.90, *p* = .1311, 95% CI [-13.54, 1.76].

Given that the Decision Agent $\times$ Decision interaction does not have a significant impact on perceived control and typicality, we rule out the possibility that these two variables mediate the relationship between the interaction and the reaction towards the decision.

**Downstream Consequences.** We used PROCESS Model 8 (Hayes, 2018; 5,000 bootstrap samples) to examine the effect of interaction between Decision Agent and Decision on organizational deviance. In the model, decision agent (0 = HR Manager, 1 = AI System) was entered as an independent variable, decision (0 = Favorable, 1 = Unfavorable) was entered as a moderator, fairness was entered as a mediator, and decision acceptance, future engagement, and organizational deviance were separately entered as a dependent variable.

***Decision Acceptance.*** As shown in Table S32, the interaction between Decision Agent Condition (0 = HR Manager, 1 = AI System) and Decision Condition (0 = Favorable, 1 = Unfavorable) significantly predicted decision acceptance through perceived fairness of the decision agent. Results on conditional indirect effects showed that there was a negative significant indirect effect through fairness in the Unfavorable Condition, but not in the Favorable Condition; when the decision was unfavorable, AI (vs. human) was perceived fairer, leading to higher decision acceptance.

***Future Engagement.*** Results showed that there was a significant effect of the interaction on future engagement through fairness (Table S33). Analyses on conditional indirect effects showed that there was a significant indirect effect through fairness in the Unfavorable Condition, but not in the Favorable Condition. In the face of unfavorable decision, perceived fairness of AI (vs. human) was higher, resulting in higher future engagement.

***Organizational Deviance.*** As shown in Table S34, the interaction between Decision Agent Condition (0 = HR Manager, 1 = AI System) and Decision Condition (0 = Favorable, 1 = Unfavorable) Condition significantly predicted organizational deviance through perceived fairness of the decision agent. Results on conditional indirect effects showed that there was a negative significant indirect effect through fairness in the Unfavorable Condition, but not in the Favorable Condition.

**Table S36**

*Supplemental Study Moderated Mediation Analyses of Decision Agent by Decision on Decision Acceptance through Fairness as a Mediator*

|  | Direct Effects on Fairness | | | | |  |
| --- | --- | --- | --- | --- | --- | --- |
|  | B | | SE | | 95% CI | |
| Decision Agent (0 = HR Manager; 1 = AI System) | -.14 | | .14 | | [-.42, .14] | |
| Decision (0 = Favorable; 1 = Unfavorable) | -1.99*** | | .15 | | [-2.28, -1.70] | |
| Decision Agent $\times$ Decision | .47* | | .20 | | [.07, .86] | |
|  | Direct Effects on Decision Acceptance | | | | |  |
|  | B | SE | | 95% CI | |  |
| Decision Agent | -.19 | .14 | | [-.47, .08] | |  |
| Decision | -1.42*** | .16 | | [-1.74, -1.10] | |  |
| Decision Agent $\times$ Decision | -.21 | .20 | | [-.60, .19] | |  |
| Fairness | .66*** | .04 | | [.58, .73] | |  |
|  | Indirect Effects on Decision Acceptance | | | | |  |
|  | B | SE | | 95% CI | |  |
| Indirect Effect through Fairness | .31 | .13 | | [.04, .57] | |  |
| Favorable Decision | -.09 | .09 | | [-.27, .09] | |  |
| Unfavorable Decision | .21 | .10 | | [.03, .40] | |  |

*Note.* ^†^p < .10; *p<.05; **p<.01; ***p<.001

**Table S37**

*Supplemental Study Moderated Mediation Analyses of Decision Agent by Decision on Future Engagement through Fairness as a Mediator*

|  | Direct Effects on Fairness | | | | |  |
| --- | --- | --- | --- | --- | --- | --- |
|  | B | | SE | | 95% CI | |
| Decision Agent (0 = HR Manager; 1 = AI System) | -.14 | | .14 | | [-.42, .14] | |
| Decision (0 = Favorable; 1 = Unfavorable) | -1.99*** | | .15 | | [-2.28, -1.70] | |
| Decision Agent $\times$ Decision | .47* | | .20 | | [.07, .86] | |
|  | Direct Effects on Future Engagement | | | | |  |
|  | B | SE | | 95% CI | |  |
| Decision Agent | -.13 | .14 | | [-.41, .16] | |  |
| Decision | -.89*** | .17 | | [-1.21, -.56] | |  |
| Decision Agent $\times$ Decision | .25 | .21 | | [-.15, .66] | |  |
| Fairness | .15*** | .04 | | [.08, .23] | |  |
|  | Indirect Effects on Future Engagement | | | | |  |
|  | B | SE | | 95% CI | |  |
| Indirect Effect through Fairness | .07 | .04 | | [.008, .15] | |  |
| Favorable Decision | -.02 | .02 | | [-.07, .02] | |  |
| Unfavorable Decision | .05 | .03 | | [.006, .11] | |  |

*Note.* ^†^p < .10; *p<.05; **p<.01; ***p<.001

**Table S38**

*Supplemental Study Moderated Mediation Analyses of Decision Agent by Decision on Organizational Deviance through Fairness as a Mediator*

|  | Direct Effects on Fairness | | | | |  |
| --- | --- | --- | --- | --- | --- | --- |
|  | B | | SE | | 95% CI | |
| Decision Agent (0 = HR Manager; 1 = AI System) | -.14 | | .14 | | [-.42, .14] | |
| Decision (0 = Favorable; 1 = Unfavorable) | -1.99*** | | .15 | | [-2.28, -1.70] | |
| Decision Agent $\times$ Decision | .47* | | .20 | | [.07, .86] | |
|  | Direct Effects on Organizational Deviance | | | | |  |
|  | B | SE | | 95% CI | |  |
| Decision Agent | .18^†^ | .11 | | [-.03, .39] | |  |
| Decision | .42*** | .12 | | [.18, .66] | |  |
| Decision Agent $\times$ Decision | -.38* | .15 | | [-.68, -.09] | |  |
| Fairness | -.07* | .03 | | [-.12, -.01] | |  |
|  | Indirect Effects on Organizational Deviance | | | | |  |
|  | B | SE | | 95% CI | |  |
| Indirect Effect through Fairness | -.03 | .02 | | [-.07, -.002] | |  |
| Favorable Decision | .009 | .01 | | [-.009, .03] | |  |
| Unfavorable Decision | -.02 | .01 | | [-.05, -.001] | |  |

*Note.* ^†^p < .10; *p<.05; **p<.01; ***p<.001

***References***

Bennett, R. J., & Robinson, S. L. (2000). Development of a measure of workplace deviance. *Journal of Applied Psychology*, *85*(3), 349–360.

Johnson, J. P., Korsgaard, M. A., & Sapienza, H. J. (2002). Perceived fairness, decision control, and commitment in international joint venture management teams. *Strategic Management Journal, 23*(12), 1141–1160.

Newman, D. T., Fast, N. J., & Harmon, D. J. (2020). When eliminating bias isn’t fair: Algorithmic reductionism and procedural justice in human resource decisions. *Organizational Behavior and Human Decision Processes*, *160*, 149–167.
